# Multiple Horizontal Gene Transfer Events and Domain Fusions Have Created Novel Regulatory and Metabolic Networks in the Oomycete Genome

**DOI:** 10.1371/journal.pone.0006133

**Published:** 2009-07-02

**Authors:** Paul Francis Morris, Laura Rose Schlosser, Katherine Diane Onasch, Tom Wittenschlaeger, Ryan Austin, Nicholas Provart

**Affiliations:** 1 Department of Biological Sciences, Bowling Green State University, Bowling Green, Ohio, United States of America; 2 Cell and Systems Biology/Centre for the Analysis of Genome Evolution and Function, University of Toronto, Toronto, Ontario, Canada; Utrecht University, Netherlands

## Abstract

Complex enzymes with multiple catalytic activities are hypothesized to have evolved from more primitive precursors. Global analysis of the *Phytophthora sojae* genome using conservative criteria for evaluation of complex proteins identified 273 novel multifunctional proteins that were also conserved in *P. ramorum*. Each of these proteins contains combinations of protein motifs that are not present in bacterial, plant, animal, or fungal genomes. A subset of these proteins were also identified in the two diatom genomes, but the majority of these proteins have formed after the split between diatoms and oomycetes. Documentation of multiple cases of domain fusions that are common to both oomycetes and diatom genomes lends additional support for the hypothesis that oomycetes and diatoms are monophyletic. Bifunctional proteins that catalyze two steps in a metabolic pathway can be used to infer the interaction of orthologous proteins that exist as separate entities in other genomes. We postulated that the novel multifunctional proteins of oomycetes could function as potential Rosetta Stones to identify interacting proteins of conserved metabolic and regulatory networks in other eukaryotic genomes. However ortholog analysis of each domain within our set of 273 multifunctional proteins against 39 sequenced bacterial and eukaryotic genomes, identified only 18 candidate Rosetta Stone proteins. Thus the majority of multifunctional proteins are not Rosetta Stones, but they may nonetheless be useful in identifying novel metabolic and regulatory networks in oomycetes. Phylogenetic analysis of all the enzymes in three pathways with one or more novel multifunctional proteins was conducted to determine the probable origins of individual enzymes. These analyses revealed multiple examples of horizontal transfer from both bacterial genomes and the photosynthetic endosymbiont in the ancestral genome of Stramenopiles. The complexity of the phylogenetic origins of these metabolic pathways and the paucity of Rosetta Stones relative to the total number of multifunctional proteins suggests that the proteome of oomycetes has few features in common with other Kingdoms.

## Introduction

*Phytophthora* species are destructive plant pathogens of a wide range of plant species. *P. sojae* is a host specific pathogen of soybeans, and protection from crop losses is mediated only by an extensive breeding program to introgress new resistance genes into soybean cultivars from wild germplasm [1]. *P. infestans* is the most serious pathogen of potatoes on a worldwide basis and control of this windborne pathogen is achieved only by a significant expenditure of pesticides [2]. While one stage of their life history includes a hyphal growth form, they are classified in the Kingdom Stramenopiles, which also include photosynthetic organisms such as brown algae and diatoms. The Stramenopiles are in turn, members of a larger group the chromalveolates, which include alveolate pathogens such as *Plasmodium falciparum* and *Toxoplasma gondii* and free-living ciliates such as *Tetrahymena thermophilia* and *Paramecium tetraurelia*. Members of this superclade are thought to have been derived from a eukaryotic protist that acquired a red algal species by endosymbiosis [3]. One of the hallmarks of the genome sequence analysis of *P. sojae* and *P. ramorum* was the finding that at least 855 of the oomycete genes had a probable cyanobacterial or red algal origin [4]. Phylogenetic analyses of several chromalaveolate genomes have now shown that endosymbiotic transfer of genes to the host nucleus has had a significant impact on the evolution of these genomes [4], [5], [6].

The completion of sequences from several protist genome projects has vastly changed our understanding of this diverse group of organisms. Domain recombination events, the lateral transfer of domains from different kingdoms, and lineage-specific expansion of particular protein families have all been identified as distinctive features of several protozoan lineages [7], [8], [9], [10], [11]. Independent phylogenetic strategies have also identified several instances of ancient horizontal transfer from bacterial genomes to stramenopiles [5], [12], [13].

Composite proteins are a common feature in eukaryotic genomes with the number of composite proteins increasing in proportion to genome size [14], [15]. In the human and mouse genomes 29% of proteins have such a structure [15]. Our examination of the *P. sojae* genome identified several multifunctional genes that appeared to have arisen from novel gene fusion events. Aside from their novelty, proteins with BLAST hits to two or more proteins in other organisms have been posited as Rosetta Stones since the fusion of two or more catalytic domains as part of a metabolic or regulatory pathway has been used to infer the association of orthologous proteins containing single domains that exist as separate entities in other genomes [16], [17], [18]. Gene fusions of enzymes in already characterized metabolic pathways such as the pentafunctional ARO1 enzyme in the aromatic amino acid biosynthetic pathway of fungi are positive examples of these kinds of associations. Gene fusion also enforces the co-regulation of two domains and co-regulated genes in multienzyme complexes. However certain domains of eukaryotic proteins are said to be “promiscuous” because they appear in combinations with other domains in proteins associated with signal transduction pathways [19]. Nonetheless, there is broad conservation of domain combinations in multifunctional (MF) proteins from sequenced genomes [20]. We hypothesized that some of the multifunctional proteins in the *P. sojae* genome would include proteins involved in metabolic or regulatory pathways that are conserved across Kingdoms. Analysis of these proteins might also reveal examples of interkingdom gene fusions [21] where one of the domains was acquired from either the ancestral photosynthetic endosymbiont or a bacterial genome. Here, we have used reciprocal shortest distance (RSD) algorithm [22] which is a conservative approach to identifying potential orthologs in 39 eukaryotic and bacterial genomes.

## Materials and Methods

### Sequence retrieval and analysis

Predicted proteins sequences of *P. sojae* were retrieved from DOE-JGI website, subjected to BLAST analysis against the nonredundant protein database, and InterProScan [23]. The output of these analyses was loaded into a MySQL database. To identify novel MF proteins, the database was first queried for protein models with multiple predicted protein domains where the length of the best BLAST hit was more than 100 amino acids smaller than the *P. sojae* model. A second independent query of protein models with two or more PFAM hits was also used to identify additional examples of novel MF proteins that were not detected in the first strategy. A total of 2236 models were subjected to manual curation using the DOE-JGI browser and other web-based tools. Proteins with any of the following characteristics were excluded from further analysis: 1. Proteins containing domains with homology to transposable elements. 2. Multi-exonic protein models without EST support unless they also met one of the following criteria: A: a single domain that spanned one or more exons. B: proteins were members of a multi-gene family in *P. sojae* in which the majority of family members contained no introns.

Draft sequences were visually inspected to remove introns not needed to maintain an open reading frame, and to identify a stop site for proteins judged to be a fusion of two adjacent proteins. Each of the domains that were identified by BLASTP analysis as showing homology to an individual protein in other species were split into separate models for RSD analysis. The majority of these novel MF proteins had BLAST hits to only two different types of proteins in other species. A subset of the MF proteins were found to have significant BLASTP hits to only one of the domains and were excluded from RSD analysis to identify Rosetta Stone candidates.

Protein sequences for *E. huxleyi* V1, *M. brevicollis* V1.0, *N. hematococca* V2, *P. ramorum* V1, *P. sojae* V1, *P. patens ssp patens* V1, *P. tricornatum* V2, *P. stipitis* V2, *T. pseudonana* V3 were downloaded from the DOE Joint Genome Initiative website at http://www.jgi.doe.gov/ (2/23/08). Protein sequences for *Aedes egyptii*, *Anopheles gambiae*, *Ashbya gossypii*, *Aspergillus fumigatus*, *Caenorhabditis briggsae*, *C. elegans*, *Candida glabrata*, *Danio rerio*, *Debaryomyces hansenii*, *Drosophila melanogaster*, *D. pseudoobscura*, *Escherichia coli*, *Kluyveromyces lactis*, *Leishmania major*, *Magnaporthe grisea*, *Mus musculus*, *Neurospora crassa*, *Phaeosphaeria nodorum*, *Plasmodium yoelli*, *Rattus norvegicus*, *Saccharomyces cerevisiae*, *Streptomyces coelicolor*, *Schizosaccharomyces pombe*, *Tetraodon nigroviridis*, and *Yersinia lipolytica* were downloaded from the Expasy website [24] at ftp://ftp.expasy.org/ (9/27/08). Protein sequences for *Gloeobacter violaceus*, *Nostoc commune*, and *Thermosynechococcus elongates w*ere downloaded from Cyanobase at http://bacteria.kazusa.or.jp/cyanobase/ (9/28/08). Protein sequences for *Arabidopsis thaliana* (TAIR8) were downloaded from http://www.arabidopsis.org/. Proteins sequences from the *Vitis vinifera* genome were downloaded from NCBI http://www.ncbi.nlm.nih.gov/sites/entrez?db=taxonomy (2/23/08). The *Cyanidioschyzon merolae* proteome was downloaded from the *C. merolae* webpage at http://merolae.biol.s.u-tokyo.ac.jp/. The *Oryza sativa* proteome (Build 3) was downloaded from the rice annotation project database website at http://rapdb.dna.affrc.go.jp/. Sequences for *Plasmodium faliciparum* were obtained from PlasmoDB http://www.plasmodb.org/plasmo/ (9/26/08).

The RSD program was downloaded and installed on a Mac desktop computer following instructions outlined in the RSD tutorial. For this analysis, the curated split models of *P. sojae* proteins were added in replacement of the identified multifunctional proteins. Predicted orthologs that were generated by RSD analysis for each split model were loaded into a Filemaker database. Potential Rosetta Stone candidates were identified by querying the database for genomes with orthologs to two or more of the split models associated with a multifunctional protein.

### Analysis of orthologs in *P. ramorum, Phaeodactorum tricornutum* and *Thalassiosira pseudonana* genomes

To determine whether domain fusion events resulting in the formation of novel MF proteins occurred prior to the split of oomycetes and diatoms, both split and complete models of *P. sojae* MF proteins were used in separate ortholog searches of the *P. ramorum* and diatom genomes. In cases where RSD analysis identified hits to only one of the domains, the *P. sojae* MF proteins were used in a TBLASTN analysis to determine if synteny of domains was retained in the assembled diatom genomes. The diatom gene model which came closest to describing the scaffold region showing homology to the *P. sojae* gene was listed as the probable ortholog.

### Analysis of sequences from Metabolic Pathways

The MetaCyc tool [25] (http://metacyc.org/) was used to identify *P. sojae* gene models from several metabolic pathways with multiple examples of novel MF proteins. Homologous sequences to *P. sojae* genes in other kingdoms were identified by BLASTP analysis of the SwissProt database and aligned using ClustalW [26]. Maximum likelihood analysis was done using PHYML v2.4.4 [27]. ML analysis was done with bootstrapping (100 replicates) using the WAG evolutionary model, assuming four substitution rate categories, and using the program defaults for estimating the gamma distribution parameter and optimization of trees. Phylogenetic trees were drawn as radial phylograms using Dendroscope [28].

## Results

The existence of novel MF proteins in the *P. sojae* genome was first observed in the course of examining protein models on the JGI Genome Browser. Several instances were noted of protein models where multiple motifs were contained within a single exon, or closely spaced exons with EST support. In each case, BLAST analysis of N-terminal and C-terminal regions gave hits to dissimilar kinds of proteins. Notable examples of such proteins included nine phosphatidylinositol-4-phosphate 5 kinases with TM domains characteristic of G-coupled receptors. A second type of fused protein involved a potassium channel that in other eukaryotes functions *in vivo* as a tetramer [29]. In the oomycetes, a single exon contains the four motifs characteristic of each monomer.

**Figure 1:**
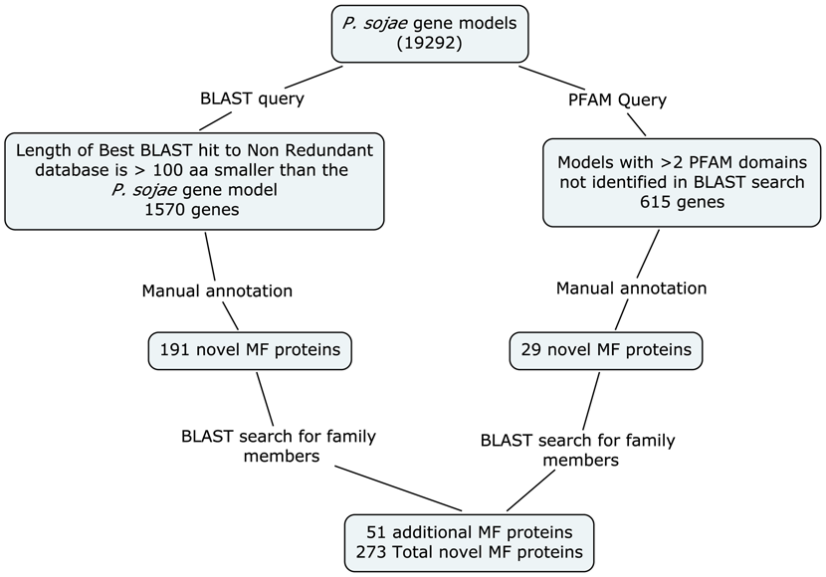
Search strategy to identify novel multifunctional proteins in the *Phytophthora sojae* genome.

A schematic outline of the strategy used to identify novel MF proteins in *P. sojae* is shown in Fig. 1. Manual annotation of 1570 *P. sojae* models that were at least 100 amino acids longer than the best BLASTP hit against the nonreduntant database identified 191 novel multifunctional proteins. An additional 29 models were identified by manual annotation of 615 models with multiple PFAM hits. Finally, 51 MF proteins were identified by serendipitous discovery or annotation of proteins that were members of a family of novel multifunctional proteins. The 273 proteins in this data set (Table S1) were assembled to test the hypothesis that the orthologs to the domains in these proteins functioned together in metabolic or regulatory networks [17], [18], [16]. An additional 130 predicted genes were excluded from this set because they may simply be two adjacent proteins that were fused into a single protein by the gene prediction software, and there was no EST support for those models. The total number of novel multifunctional proteins is expected to increase as refinements to gene prediction software and additional EST data identifies both multi-exonic proteins and adjacent genes on the genome as single gene models. Novel MF proteins are conserved in the *P. ramorum* genome. Using the curated set of 273 *P. sojae* proteins, RSD analysis identified 210 orthologous proteins in *P. ramorum*, and BLAST analysis and visual inspection of the *P. ramorum* genome to check for synteny using the DOE-JGI browser confirmed the presence of *P. ramorum* orthologs for all 273 proteins (Table S1).

Based on visual inspection of domain descriptions, 47 of the 273 gene models are derived from gene fusions of homologous domains. The largest family of such proteins consists of nine tetrameric calcium-activated potassium channels. MF proteins were catalogued as metabolic if one or more of the domains were associated with metabolism. All remaining MF proteins had domains associated with ion transport, signaling, or protein-protein interactions, and were catalogued as involved in cellular regulation. Sixty three MF proteins have motifs suggesting that they function in metabolic pathways. Thus the majority of novel MF proteins have functional domains that are typically associated with proteins in signaling pathways.

### RSD analysis

RSD analysis has identified 18 potential Rosetta Stones with interactors in 39 different species representing organisms from bacteria, plant, fungal, and animal kingdoms (Table S2). The complete list of orthologs to each of the MF proteins is given in Table S3. Five Rosetta Stones identified predicted interacting proteins in the human genome, but none of these are presently supported by experimental data. (http://hwiki.fzk.de/wiki/index.php/Main_Page). Five Rosetta Stones are MF proteins that catalyze consecutive steps in well-characterized metabolic pathways. These include Ps112102, which has protein domains defining catalytic activities for the first three steps in sulfate assimilation (orthologs in 10 genomes) and Ps131776 which has domains with predicted glycerol acyltransferase and glycerol acyl reductase activities (orthologs in11 genomes). The human orthologs of Ps131776 are localized in the peroxisome and the ortholog to the N terminal domain is required for the conversion of fatty acids to fatty alcohols [30]. Plants contain homologs to only the first domain of this MF protein and orthologs to it were identified in *A. thaliana*, *O. sativa* and *P. patens*. Expression of this gene in *A. thaliana* is critical for the development of a normal pollen cell wall [31]. While the majority of novel MF proteins in *P. sojae* contain domains characteristic of proteins associated with scaffold or regulatory pathways, RSD analysis identified only nine candidate Rosetta Stones with domains associated with regulatory pathways. The majority of these proteins identified orthologs in only one or two species.

Since our set of novel MF proteins included 63 examples with domains associated with metabolism, we next sought to determine if this level of gene fusion in metabolic pathways was typical for other eukaryotic genomes. A recent survey of plant genomes identified only 22 bifunctional proteins [32] in metabolic pathways that are also present in oomycetes, and there are *P. sojae* models with overlapping metabolic activities for 19 of these plant enzymes. *P. sojae* also contains homologs for 20 of the 29 human fusion proteins of prokaryotic origins [33]. Table 1 lists 39 bifunctional enzymes in *P. sojae* that have homologs in plant, fungal, or human genomes. The presence of these bifunctional enzymes in multiple kingdoms may be indicative of either ancient gene fusions in a common eukaryotic ancestor or independent fusion events. However Ps135354 is a clear example of an independent fusion event. The fusion of orotate phosphoribosyltransferase (OPRT) and orotdine 5′ monophosphate decarboxylase (OMPDC), the last two steps in the pyrimidine biosynthetic pathway has the domain order OPRT-OMPDC in plants and metazoa, and OMPDC-OPRT in oomycetes, diatoms, kinetoplastids and some cyanobacteria [34]. Phylogenetic analysis indicated these novel gene fusions to be independent evolutionary events [34], and our analysis of the two *Phytophthora* sequences along with those of the diatom genomes supported those conclusions (not shown).

**Table 1:** Multifunctional metabolic enzymes in oomycete and other eukaryotic genomes.

Gene_ID	Metabolic Process	Catalytic activities	*A. thaliana*	*H. sapiens*	*S. cerevisiae*
Ps108209	amino acid catabolism	Methyl malonyl Coenzyme A mutase	none	P22033	none
Ps108248	aspartate metabolism	Aspartate kinase-homoserine dehydrogenase	Q9SA18	none	none
Ps108383	proline synthesis	Delta 1-pyrroline-5-carboxylate synthetase (P5CS)	P54887	none	P54885
Ps108492	purine biosynthesis	AIR synthase related protein	A9Y5J1	A8K8N7	Q45U25
Ps108690	glutathione	1-cys-peroxiredoxin	O04005	P30041	P34227
Ps108696	tryptophan	Anthranilate synthase component II	Q9LUB2	None	P00937
Ps108681	fucose	GDP-L-fucose synthase	O49213	Q13630	none
Ps108780	glycolysis	Pyruvate carboxylase	none	P11498	P32327
Ps108801	lysine catabolism	Lysine-ketoglutarate reductase-saccharopine dehydrogenase	Q9SMZ4	Q9UDR5	P38999
Ps108913	Lipid biosynthesis	ATP citrate lyase	none	P53396	none
Ps108918		Acetyl CoA carboxylase	Q38970	Q13085	P32874
Ps108989	mRNA synthesis	RNA polymerase II	P18616	P24928	P04050
Ps109060	histidine synthesis	Glutamine amidotransferase glutamine cyclase	Q9SZ30	none	P33734
Ps109110	riboflavin metabolism	GTP cyclohydrolase II Dihydropteroate synthase	Q9SJY9	none	Q99258
Ps109141	folic acid	7,8-Dihydro-6-hydroxymethylpterin-pyrophosphokinase,	Q9SZV3	none	P53848
Ps109146	Mo cofactor biosynthesis	Molybdenum cofactor biosynthesis protein 1	Q39055	Q9NZB8-5	none
Ps109150	ketone metabolism	Succinyl-CoA:3-ketoacid-coenzyme A transferase	none	P55809	none
Ps109206	urea cycle	Carbamoyl-phosphate synthetase III;	Q9SZV3	none	Q2VQX0
Ps109271	mRNA synthesis	RNA polymerase	P38420	B4DHJ3	P08518
Ps109291	fatty acid degradation	Enoyl-CoA hydratase; Long chain 3-hydroxyacyl-CoA dehydrogenase	Q9ZPI6	P40939	P28817
Ps109321	tetrahydrofolate	Dihydrofolate reductase-thymidylate synthase	Q05762	P04818	None
Ps109425	urea cycle	Carbamoyl phosphate synthetase	Q42601	P31327	PO7259
Ps109486	purine biosynthesis	Phosphoribosylaminoimidazole carboxamide formyltransferase activity	Q8RWT5	P31939	P54113
Ps109918	fatty acid metabolism	Acetyl-coenzyme A carboxylase	Q84MM8	none	P36022
Ps123952	PABA	p-aminobenzoate synthase-glutamine-amidotransferase	Q9ZV26	P49915	P49915
Ps128107	Mo cofactor biosynthesis	Molybdopterin biosynthesis	Q39054	Q9NQX3	none
Ps129332	adenine biosynthesis	ADE1	P52420	Q59HH3	P07244
Ps131873	glucose metabolism	Phosphofructo-2 kinase, fructose 2,6 bisphosphatase	Q9MB58	Q16875	P32604
Ps133964	multi-functional tRNA	Glutamyl prolyl tRNA synthetase	O82462	Q8NAJ6	P46655
Ps134345	biotin synthesis	Bifunctional diaminopelargonate synthase-dethiobiotin synthetase	B0F481	none	none
Ps134889	sucrose metabolism	Phosphofructo-2 kinase, fructose 2,6 bisphosphatase	Q9MB58	B2R6L2	P40433
Ps135279	histidine biosynthesis	Phosphoribosyl-ATP pyrophosphohydrolase–Phosphoribosyl-AMP cyclohydrolase	novel	novel	novel
Ps135354	pyrimidine metabolism	Orotidine 5′phosphate decarboxylase-Orotate phosphoribosyltransferase	none	none	none
Ps136410	Aspartate biosynthesis	Aspartate kinase-homoserine dehydrogenase	Q9SA18	None	P10869
Ps137041	RNA polymerase	DNA-directed RNA polymerase III	Q9LVH0	O14802	P040051
Ps137320	Topoisomerase/gyrase	Topoisomerase/gyrase	P30182	P11388	Q8TG58
Ps141174	glucose metabolism	Phosphofructo-2 kinase, fructose 2,6 bisphosphatase	Q9MB58	Q16877	P32604
Ps143339	amino acid biosynthesis	Pentafunctional aromatic amino acid synthesis	None	None	P08566
Ps155429	Aspartate biosynthesis	Aspartate kinase-homoserine dehydrogenase	Q9SA18	None	P10869

### Formation of some novel MF proteins preceded the split between diatoms and oomycetes

To determine if the formation of MF proteins was initiated prior to the split between diatoms and oomycetes, we analyzed the diatom genomes for orthologous sequences to the *P. sojae* MF proteins. Table 2 shows 21 examples where synteny of domain order has been retained in the diatom and oomycete genomes. Since these proteins represent a small fraction of the 273 novel MF proteins in this data set, the majority of gene fusion events that resulted in the formation of these proteins in the oomycete genomes occurred after the split between diatoms and oomycetes.

**Table 2:** Novel multifunctional proteins present in the *P. sojae*, *P. tricornutum* and *T. pseudonana* genomes.

*P. sojae*	*T. pseudonana*	*P. tricornutum*	Domains
Ps108305	Tp28241	32747	Glyceraldehyde 3-phosphate dehydrogenase
Ps109321	Tp13647	1690	bifunctional dihydrofolate reductase-thymidylate synthase
Ps110986	Tp268564	no model in PT	Novel
Ps112102	Tp35055	19901	Adenylylsulfate kinase
Ps116824	Tp5802	8996	N terminal protein is upstream 5801
Ps127510	Tp8169	39696	calmodulin and leucine rich repeats domains not present
Ps128154	Tp260768	16649	Beta-ketoacyl synthase &hydroxymethylglutaryl-CoA synthase
Ps129073	Tp35637	40689	Phosphodiesterase and phosphohydrolase
Ps129281	Tp8768	no	RNA Bindi55ng
Ps129631	Tp21612	21612	Hypothetical protein
Ps131310	Tp21710	no blast hit	ankyrin
Ps131558	Tp264628		Mannitol dehydrogenase
Ps132073	Tp24275	47805	Heavy metal transport
Ps132790	Tp3330	3120	Cyclinc nucleotide binding, regulator of G protein
Ps134136	Tp262528	39951	Phosphatidylinositol 3- and 4-kinase, plekstrin
Ps134555	Tp26966	36588	Mov 3 family PWWP domain
Ps135354	Tp37071	11740	Pyrimidine metabolism
Ps138204	Tp12191	no direct hit	Hypothetical protein
Ps138371	Tp263580	48692	Cold shock protein
Ps138788	Tp264631	48208	Cyclin, N-terminal
Ps140109	Tp262068	No direct hit	Plekstrin, Zn-finger

**Table 3:** Phylogenetic associations of enzyme domains in serine biosynthesis and sulfate assimilation pathways of *P. sojae* and *P. ramorum*.

Process	Gene Function	GENE_ID*	Clade Association
Serine Biosynthesis			
	1 phosphoglycerate dehydrogenase	Ps142688a	metazoan
	2 phosphoserine aminotransferase	Ps142688b	bacterial
	3 phosphoserine phosphatase	Ps132144 Ps134157 Ps157034	bacterial
	4 serine-pyruvate aminotransferase	Ps109249	bacterial
Sulfate Assimilation/Metabolism			
	1 Adenyl sulfate kinase	Ps112102a	indeterminant
	2 ATP sulfuryase	Ps112102b	indeterminant
	3 pyrrophosphatase	Ps112102c	indeterminant
	4 phosphoadenosine phosphosulfate reductase	Ps156997a	plant/cyano
	5 glutaredoxin	Ps156997b	bacterial
	6 sulfite reductase ECM17	Ps139493	fungal
	6 sulfite reductase MET10	Ps139488	fungal
	7 cysteine synthase	Ps109172, Ps109175	plant
	8 cystathionine gamma lyase	Ps109222	metazoan
	9 cystathionine beta lyase	Ps120833	indeterminant

* Letter following Gene ID indicates a multifunctional protein where each of the domains was analyzed separately.

Clade association was assigned when bootstrap support was >70% of 100 replicates using PHYML parameters as described in methods.

### Lysine biosynthesis

**Figure 2:**
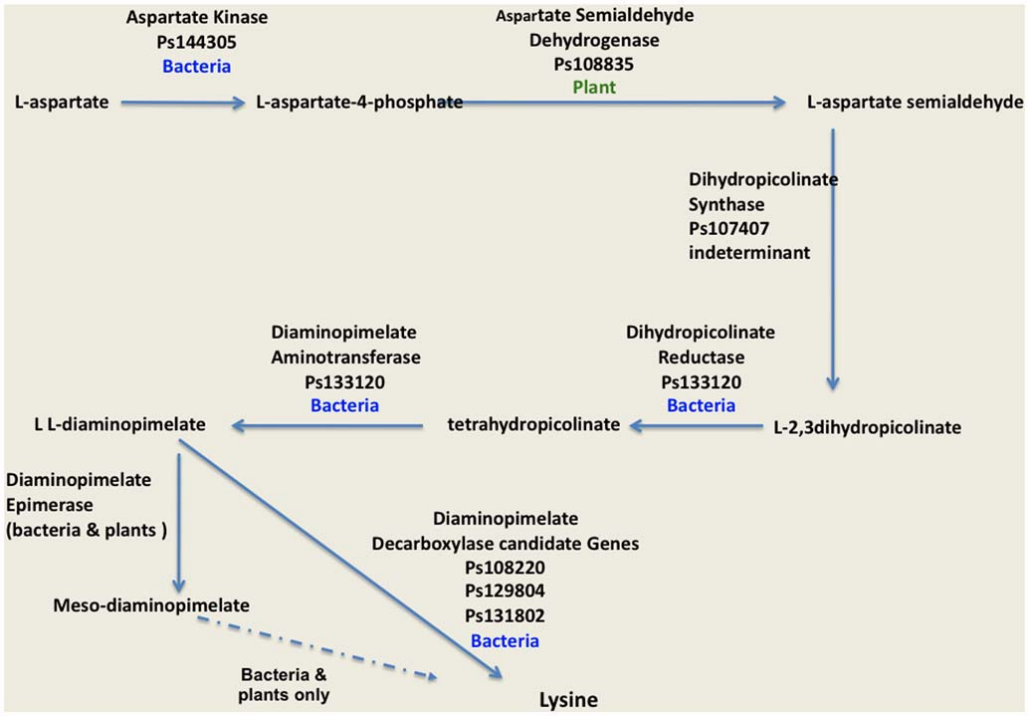
Lysine biosynthetic pathway of oomycetes. Gene model IDs for *P. sojae* are shown for each step on the pathway. The probable origin of each gene model based on PHYML analysis is listed as bacterial, metazoan, plant or unknown. The oomycete pathway closely mirrors the plant biosynthetic strategy, but has one fewer step and the majority of enzymes appear to be of bacterial origin.

The MetaCyc pathway database presently lists six biochemical strategies for the synthesis of lysine, a clear indication that multiple, distinct biosynthetic strategies are possible [25]. In oomycetes, synthesis of lysine is carried out by five enzymes that catalyze six enzymatic reactions (Fig. 2). Mechanistically, the biosynthetic strategy of oomycetes follows that of plants, although, phylogenetic analysis shows that the oomycete sequences cluster with sequences from bacterial or archaeal domains of life. Analysis of the two diatom genomes indicates that they also follow the same enzymatic strategy as oomycetes, although only some of diatom genes share the same phylogenetic origins with oomycetes.

Phylogenetic analysis of aspartate kinase, the first enzyme in this pathway shows that while the diatom sequences group with those from plant genomes, oomycete sequences are more closely related to bacterial bifunctional enzymes with diaminopimelate decarboxylase and aspartate kinase activity (Fig. S1). Phylogenetic analysis of aspartate semialdehyde dehydrogenase, the second step in this pathway show that both diatoms and oomycete genes are grouped together in the same clade with strong bootstrap support, and are distinct from a clade of cyanobacterial and plant sequences. However the Stramenopile sequences also cluster with strong bootstrap support to sequences from three archaea genomes (Fig. S2). Perhaps the simplest explanation for the grouping of archaea genes with stramenopiles would be a horizontal transfer to archaea from the ancestral bacterial genome that also transferred this gene to Stramenopiles. Phylogenetic analysis of dihydropicolinate synthase, the third enzyme in the oomycete lysine biosynthetic pathway, indicates that while the oomycete sequences are clustered away from other eukaryotic genomes, they do not form a strongly supported cluster with bacterial sequences (Fig. S3). The next two steps in the pathway are catalyzed by a unique fused protein with dihydropicolinate reductase and diaminopimelate aminotransferase activities. These domains have close homologs in only bacterial genomes (Fig. S4–Fig. S5).

**Figure 3:**
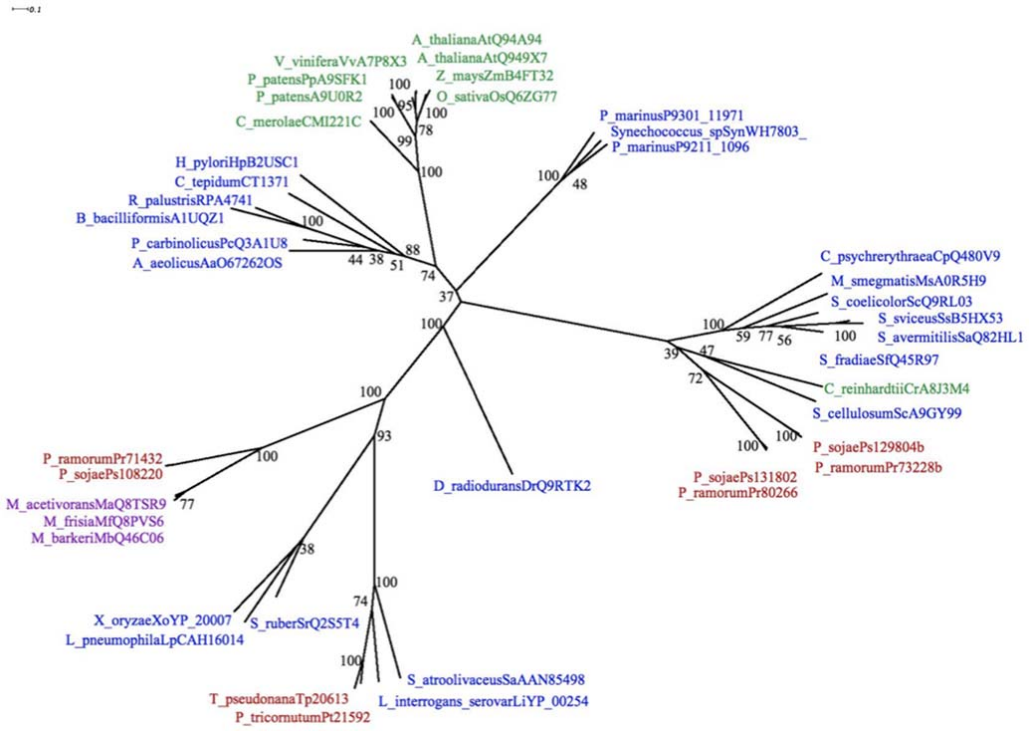
PHYML analysis of genes with homology to oomycete genes with predicted domains for arginine, ornithine or diaminopimelate decarboxylase activity. Numbers at nodes indicate bootstrap support values (100 replications). Gene names are color coded to indicate the major phylogenetic groups: archaea, purple; bacteria, blue; plants, green; stramenopiles, red.

In bacteria, the conversion of L,L diaminopimelate to meso-diaminopimelate by epimerase is a necessary step because meso-diaminopimelate is an essential component of the bacterial peptidoglycan cell wall. Plants and diatoms also have an epimerase and, while they do not make peptidoglycans, nine homologous genes to bacterial homologs of the peptidoglycan pathway play an important role in chloroplast cell division in mosses and potentially other plants [35]. We searched *P. ramorum*, *P. sojae* and *P. infestans* genomes using plant and bacterial epimerases in a TBLASTN search and found no evidence for epimerase activity. Thus we postulate that oomycetes convert L- diaminopimelate directly to lysine without an intermediate step. The oomycete genomes each contain three candidate genes with PFAM domains for ornithinine, diaminopimelate, or arginine (ORN/DAP/ARG) decarboxylase activity, suggesting that one or more of these proteins function as diaminopimelate decarboxylase, the last step in the pathway. Two of these proteins in each of the oomycete genomes cluster with sequences from bacterial genomes (Fig. 3). The third pair of genes (Ps108220 and Pr71432) is in a separate clade of diverse bacterial sequences, but the oomycete sequences form a cluster with sequences from Archaea genomes (Fig. 3). The clustering of archaeal sequences in this clade of bacterial and oomycete genes is suggestive of a horizontal transfer to archaea from the ancestral bacterial genome that also transferred this domain to oomycetes. Notably, this clade also includes the bacterial bifunctional proteins (aspartate kinase-diaminopimelate decarboxylase) from *X. oryzae, L. pneumophila* and *S. ruber*. The N terminal domain of these bacterial genes also clustered with oomycete genes with predicted aspartate kinase activity (Fig. S1). The N-terminal domain of the *P. sojae* gene Ps128804 and its ortholog in *P. ramorum* Pr72228 is a conserved domain of unknown function that clusters with sequences from bacterial genomes (Fig. 3).

### Serine biosynthesis

One of the potential serine biosynthetic strategies of oomycetes involves the reduction of phosphoglycerate to make 3-phosphohydroxypyruvate, a phosphoserine aminotransferase to make phosphoserine, and a phosphoserine phosphatase to produce serine. In oomycetes, the first two steps in this pathway are carried out by a bifunctional enzyme (Ps142688 and Pr80380). Phylogenetic analysis of the N terminal domain shows that the oomycete domains cluster with genes from metazoans (Fig. S7). Phylogenetic analysis of both the C-terminal domain of the bifunctional enzyme, and the oomycete phosphoserine phosphatase gene show strong support for a bacterial origin of these genes (Figs. S8–S9). An alternate biosynthetic pathway leading to serine could involve the amination of glyoxylate, but in this case, too, there is one aminotransferase enzyme in each genome (Ps109249 and Pr77437) and phylogenetic analysis suggests that these proteins were acquired by an ancient horizontal transfer event from a bacterial genome (Fig. S10).

### Sulfate assimilation

Phylogenetic analysis of the sulfate assimilation pathway in eukaryotes has indicated a very complex pattern of inheritance across the Kingdoms with multiple examples of gene fusions and horizontal transfer events (Patron et al. 2008). In fungi and plants, the reduction of sulfate and the subsequent transfer of the sulfhydryl group to O-acetyl-serine resulting in the formation of cysteine is mediated by six enzymes. In animals, the first two enzymes in this pathway, ATP surfurylase and adenylsulfate kinase, form a single bifunctional enzyme. These two functional domains are present in the oomycete models but the oomycete proteins also contain an additional pyrophosphatase domain to break down the pyrophosphate that is produced as a byproduct of ATP surfurylase. Phylogenetic analysis suggests that the fusion of the three domains is an ancient event since it is also present in the two diatom genomes as well as the coccolithophore *Emiliania huxleyi*, and separate analysis of each of the domains show that they cluster in the same clades (Figs. S11, S12, S13).

The second oomycete gene in this pathway (Ps156997) clusters in a clade with cyanobacterial sequences, while higher plant sequences cluster in a separate clade and the diatom gene form a clade with moss and Selaginella genes (Fig. S14). The oomycete genes also contain an additional glutaredoxin motif that phylogenetic analysis indicates is of probable bacterial origin (Fig. S15). The *P. sojae* models Ps139493 and Ps139488 are homologous to the *S. cerevisiae* enzymes ECM17 and MET10 that carry out the reduction of sulfite to hydrogen sulfide (Fig. S16–17). *P. sojae* contains two cysteine synthases (Ps109172 and Ps109755) that are phylogenetically related to the plant cytosolic enzymes (Fig. S18). In oomycetes, the conversion of cystathionine to cystein by cystathionine gamma lyase is done by an enzyme that clusters with proteins from metzoans (Fig. S19). Finally the conversiton of cystathionine to homocysteine by cystathionine B lyase is achieved by an enzyme that clustered with both plant and fungal proteins (Fig. S20).

In summary the contribution of horizontal transfer from both the algal endosymbiont and bacterial genomes have enabled oomycete and diatoms to develop unique metabolic networks.

## Discussion

The large number of both regulatory and metabolic proteins in the oomycete genomes that are derived from novel gene fusion events emphasizes the distinctive nature of these genomes. An important feature of the oomycete genomes that aided in the identification of verifiable novel MF proteins is that the majority of protein models are defined by a single open reading frame. We noted 100% conservation of the 273 MF proteins between *P. sojae* and *P. ramorum*. Extending the analysis of novel MF proteins to other oomycete genomes will enable us to address several questions. What is the level of conservation of these proteins? Does domain fusion and rearrangement continue to be an important factor in contributing to species diversity? What is the role of intron gain or loss in the evolution of these proteins? A subset of the MF proteins described in this study are also present in the two diatom genomes, a likely indication that some of these fusion events preceded the split between diatoms and oomycetes (Table 2). Gene islands are also a feature of the *P. tricornutum* and *T. pseudonana* genomes, and both species have small introns. The diatom genomes have proven to be surprisingly diverse but the contribution of novel gene fusions to that diversity has not been specifically addressed [5]. Thus, in addition to the set of MF proteins that are conserved between the diatom and oomycete genomes, the diatom genome may also include several novel MF proteins that are presently recognized as separate proteins, due to limitations in gene prediction software.

The majority of *P. sojae* MF proteins analyzed here contain domains such as calcium binding, protein binding, Zn finger or kinase networks, consistent with their role in regulatory networks. For most proteins, RSD analysis identified orthologs to only one of the domains in each MF protein (Table S2). Since the complete proteins have no direct orthologs in other eukaryotic genomes, each of the domains may interact with other proteins to form novel oomycete regulatory networks. Since these proteins are conserved in *P. ramorum* and presumably other oomycete genomes, they may also serve to identify some of the novel regulatory networks in this important group of plant pathogens.

A particularly striking feature of the oomycete genome is the unusual number of gene fusion events in primary metabolic pathways. The oomycetes have acquired the majority of bifunctional metabolic enzymes in metabolic pathways that they share with plant, fungal and animal kingdoms (Table 1), along with 63 additional models that were identified in this survey. What conditions in the evolutionary history of oomycetes drove the selection of so many gene fusion events in common metabolic pathways? Biochemical studies suggest that the enzymes involved in many metabolic processes form large macromolecular complexes [36], [37] and fusion of catalytic domains represents a rare, more constrained version of such complexes. Gene fusions may further increase the efficiency of the biosynthetic process by enabling metabolic channeling between catalytic domains. However if the driving force for such events in eukaryotic genomes was simply a matter of metabolic efficiency, the existence of bifunctional metabolic proteins would be much more widespread. Selection pressure that favors fusion events of metabolic genes must balance metabolic throughput with the need to regulate these processes. While metabolic channeling can serve to improve the catalytic efficiency of the complex, transient protein-protein interactions can function to regulate the catalytic activity of other enzymes in the complex [37]. In this regard, biochemical analysis of a bifunctional enzyme in the lysine degradation pathway of *A. thaliana* serves to illustrate the importance of protein-protein binding in metabolic pathways. The bifunctional LKR/SDH locus also codes for monofunctional enzymes with lysine-glutarate reductase and saccharopine reductase activities, and the monofunctional enzymes have strikingly different catalytic activities than the fused model [38].

An alternative hypothesis is that the processes of gene duplication and sequence divergence in plant, fungal, and metazoan genomes did not produce the kinds of combinations needed to enable the domains to fuse and still retain optimal catalytic activity. Cross genomic comparisons of the enzymes in the pyrimidine biosynthetic and sulfate assimilation pathways provided multiple examples of independent gene fusion events [34], [39]. However, the absence of fusion events in most phylogenetic groups may hint that the domains of genes that catalyzed individual steps in these pathways were not sufficiently compatible to enable gene fusion to be selected for. Given this scenario, we would expect a similar high rate of domain fusion events in diatom genomes since they share a similar evolutionary history including the acquisition of bacterial genes [5]. The analysis of point mutations in the MF proteins across multiple oomycetes genomes may prove to be a useful means of evaluating the relative importance of catalytic efficiency and regulatory control in selected metabolic pathways.

**Figure 4:**
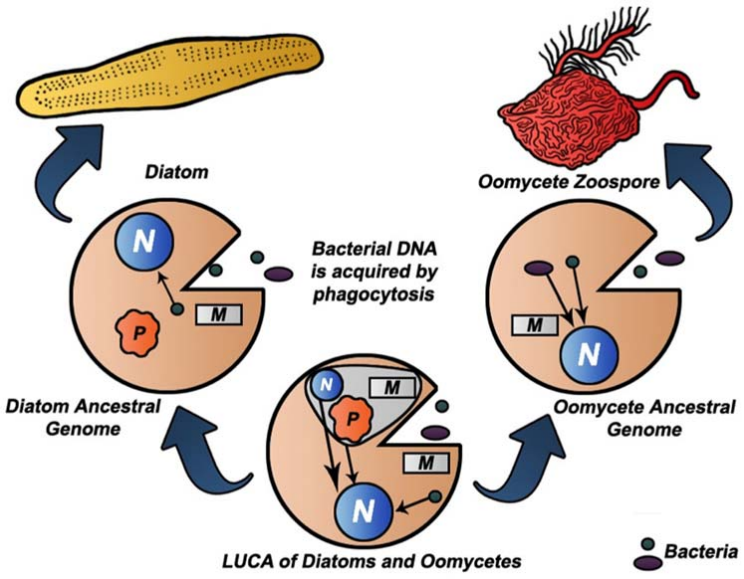
Divergence of diatoms and oomycetes from a common ancestral genome. Selective transfer of DNA from the endosymbiont of LUCA, and the continued acquisition of bacterial DNA by phagocytosis after the separation of oomycetes and diatoms has contributed to the divergence of these genomes.

Lateral transfer appears to played a major role in the evolution of proteins in Bacteria and Archaea [40], [41], [42]. Phylogenetic analyses now suggest that horizontal gene transfer has played a significant role in evolutionary biology of eukaryotes, and particularly protistan lineages [5], [10], [12], [13], [39], [43]. That some of the MF proteins contained one or more domains with the lowest RSD scores coming from plant genomes was not surprising, since global analysis of the *P. sojae* and *P. ramorum* genomes identified 855 genes of probable origin from the photosynthetic endosymbiont to the ancestral genome of oomycetes. The presence of bacterial domains in the MF proteins was unexpected, since we did not did not systematically search for such proteins. Here we have described a significant contribution of bacterial domains, both as single entities, and as components of novel MF proteins in two metabolic pathways. Our observations extend those of other recent reports documenting the lateral transfer of bacterial genes into oomycete genomes [5], [13]. The model shown in Fig. 4 is intended to illustrate that the processes of horizontal transfer of genes from the photosynthetic endosymbiont, along with the independent acquisition of genes from bacterial genomes continued after the split of these phylogenetic lineages and may help to account for the surprising level of diversity between diatoms and oomycetes. One measure of this diversity can be seen in the phylogenetic analysis of serine and lysine biosynthesis. Oomycetes have adopted two biosynthetic strategies for serine biosynthesis from bacteria. While both diatoms and oomycetes share a similar biosynthetic strategy for lysine, in several cases the diatoms' genes were clustered in different clades (Fig. S1, S3 S5).

Lateral transfer of domains associated with signaling proteins from both bacterial and host genomes of various protist genomes has been cited as evidence of the diversification of regulatory networks in several protist genomes [11]. In *P. sojae*, the curated set of MF proteins included 171 models with two or more domains that are typically associated with signaling networks. These proteins represent nodal proteins in novel oomycete regulatory pathways. One of the predictions made to support the investment in whole genome sequencing project of oomycete pathogens was that this data would enable the identification of new targets of pesticides. An integrated approach incorporating expression analyses, proteomics, and comparative genomics is now needed to define the novel proteome of oomycetes, so that we can identify both the unique growth and pathogenic strategies of this group of pathogens.

## Supporting Information

Table S1 Novel Multifunctional Proteins of P. sojae and P. ramorum. This file contains a list of the multifunctional proteins from P sojae, and their ortholog from P ramorum. the file also contains a description of the domains of these proteins and the amino acid sequence of the protein domains that were subjected to ortholog analysis by reciprocal smallest distance.splits
(0.05 MB XLS)

Table S2 Candidate Rosetta Stone proteins in the P sojae genome as identified by RSD analysis. This table identifies all the multifunctional gene models in P sojae that had orthologs to at least two of their domains in one or more species
(0.16 MB DOC)

Table S3 List of orthologs to the multifunctional protein domains of P. sojae. This table lists all the orthologs from 39 species to the individual domains of the multifunctional proteins
(0.13 MB XLS)

Figure S1 PHYML analysis of genes with homology to the aspartate kinase domain of Ps144305. The P. sojae enzyme is the first committed step in a predicted lysine biosynthetic pathway of oomycetes. Numbers at nodes indicate bootstrap support values (100 replications). Gene names are color coded to indicate the major phylogenetic groups: archaea, purple; bacteria, blue; opistokonts, brown; plants, green; stramenopiles, red.
(3.00 MB TIF)

Figure S2 PHYML analysis of genes with homology to the aspartate semi-aldehyde dehydrogenase domain of Ps108835. The P. sojae enzyme is the second step a predicted lysine biosynthetic pathway of oomycetes. Numbers at nodes indicate bootstrap support values (100 replications). Gene names are color coded to indicate the major phylogenetic groups: archaea, purple; bacteria, blue; opistokonts, brown; plants, green; stramenopiles, red.
(3.00 MB TIF)

Figure S3 PHYML analysis of genes with homology to the dihydropicolinate synthase domain of Ps108407. The P. sojae enzyme is the third step in a predicted lysine biosynthetic pathway of oomycetes. Numbers at nodes indicate bootstrap support values (100 replications). Gene names are color coded to indicate the major phylogenetic groups: archaea, purple; bacteria, blue; opistokonts, brown; plants, green; stramenopiles, red.
(3.00 MB TIF)

Figure S4 PHYML analysis of genes with homology to the dihydropicolinate reductase domain of Ps133120. The P sojae enzyme carries out step four and step five of a predicted lysine biosynthetic pathway of oomycetes. Numbers at nodes indicate bootstrap support values (100 replications). Gene names are color coded to indicate the major phylogenetic groups: archaea, purple; bacteria, blue; opistokonts, brown; plants, green; stramenopiles, red.
(3.00 MB TIF)

Figure S5 PHYML analysis of genes with homology to the diaminopimelate aminotransferase domain of Ps133120. The P sojae enzyme carries out step four and step five of a predicted lysine biosynthetic pathway of oomycetes. Numbers at nodes indicate bootstrap support values (100 replications). Gene names are color coded to indicate the major phylogenetic groups: archaea, purple; bacteria, blue; opistokonts, brown; plants, green; stramenopiles, red.
(3.00 MB TIF)

Figure S6 PHYML analysis of genes with homology to the N-terminal domain of Ps129804. Numbers at nodes indicate bootstrap support values (100 replications). Gene names are color coded to indicate the major phylogenetic groups: archaea, purple; bacteria, blue; opistokonts, brown; plants, green; stramenopiles, red
(3.00 MB TIF)

Figure S7 PHYML analysis of genes with homology to the phosphoglycerate dehydrogenase domain of Ps142688. The P. sojae enzyme contains domains which catalyze the first two steps in a serine biosynthetic pathway. Numbers at nodes indicate bootstrap support values (100 replications). Gene names are color coded to indicate the major phylogenetic groups: archaea, purple; bacteria, blue; opistokonts, brown; plants, green; stramenopiles, red.
(3.00 MB TIF)

Figure S8 PHYML analysis of genes with homology to the phosphoserine aminotransferase domain of Ps142688. The P. sojae enzyme catalyzes the first two steps in a serine biosynthetic pathway. Numbers at nodes indicate bootstrap support values (100 replications). Note that the diatom genomes contain agene model in the same clade as oomycete genomes. Gene names are color coded to indicate the major phylogenetic groups: archaea, purple; bacteria, blue; opistokonts, brown; plants, green; stramenopiles, red.
(3.00 MB TIF)

Figure S9 PHYML analysis of genes with homology to the phosphoserine phosphatase domians of Ps132144 Ps134157, and Ps157034. The P. sojae enzymes catalyze the last step in a serine biosynthetic pathway. Numbers at nodes indicate bootstrap support values (100 replications). Gene names are color coded to indicate the major phylogenetic groups: archaea, purple; bacteria, blue; opistokonts, brown; plants, green; stramenopiles, red.
(3.00 MB TIF)

Figure S10 PHYML analysis of genes with homology to the serine-pyruvate aminotransferase domain of P sojae. The P. sojae enzyme catalyzes the synthesis of serine from pyruvate, a biosynthetic strategy independent of the P. sojae enzymes analyzed in Figs. S7, S8, S9. Numbers at nodes indicate bootstrap support values (100 replications). Gene names are color coded to indicate the major phylogenetic groups: archaea, purple; bacteria, blue; opistokonts, brown; plants, green; stramenopiles, red.
(3.00 MB TIF)

Figure S11 PHYML analysis of genes with homology to the adenylsulfate kinase domain of Ps112102. Numbers at nodes indicate bootstrap support values (100 replications). The oomycete, diatoms and E. huxleyi genomes contain a single protein with adenylsulfate kinase, ATP sulfurylase and pyrrophosphatase domains. The domains of these proteins have been subjected to separate phylogenetic analyses to determine whether they share a common phylogenetic origin. Gene names are color coded to indicate the major phylogenetic groups: archaea, purple; bacteria, blue; opistokonts, brown; plants, green; stramenopiles, red.
(3.00 MB TIF)

Figure S12 PHYML analysis of genes with homology to the ATP sulfurylase domain of Ps112102. Numbers at nodes indicate bootstrap support values (100 replications). The oomycete, diatoms and E. huxleyi genomes contain a single protein with adenylsulfate kinase, ATP sulfurylase and pyrrophosphatase domains. The domains of these proteins have been subjected to separate phylogenetic analyses to determine whether they share a common phylogenetic origin. Gene names are color coded to indicate the major phylogenetic groups: archaea, purple; bacteria, blue; opistokonts, brown; plants, green; stramenopiles, red.
(3.00 MB TIF)

Figure S13 PHYML analysis of genes with homology to the pyrrophosphatase domain of Ps112102. Numbers at nodes indicate bootstrap support values (100 replications). The oomycete, diatoms and E. huxleyi genomes contain a single protein with adenylsulfate kinase, ATP sulfurylase and pyrrophosphatase domains. The domains of these proteins have been subjected to separate phylogenetic analyses to determine whether they share a common phylogenetic origin. Gene names are color coded to indicate the major phylogenetic groups: archaea, purple; bacteria, blue; opistokonts, brown; plants, green; stramenopiles, red.
(3.00 MB TIF)

Figure S14 PHYML analysis of genes with homology to the phosphoadenosine phosphosulfate reductase domain of Ps156997. The P sojae enzyme catalyzes step four in the sulfate assimilation pathway. Numbers at nodes indicate bootstrap support values (100 replications). Gene names are color coded to indicate the major phylogenetic groups: archaea, purple; bacteria, blue; opistokonts, brown; plants, green; stramenopiles, red.
(3.00 MB TIF)

Figure S15 PHYML analysis of genes with homology to a novel glutaredoxin domain on Ps156997. Numbers at nodes indicate bootstrap support values (100 replications). Gene names are color coded to indicate the major phylogenetic groups: archaea, purple; bacteria, blue; opistokonts, brown; plants, green; stramenopiles, red.
(3.00 MB TIF)

Figure S16 PHYML analysis of genes with homology to Ps139493. The P. sojae enzyme is predicted to form a complex with Ps139488 to catalyze the reduction of sulfite. Numbers at nodes indicate bootstrap support values (100 replications). Gene names are color coded to indicate the major phylogenetic groups: archaea, purple; bacteria, blue; opistokonts, brown; plants, green; stramenopiles, red.
(3.00 MB TIF)

Figure S17 PHYML analysis of genes with homology to Ps139488. The P. sojae enzyme is predicted to form a complex with Ps139493 to catalyze the reduction of sulfite. Numbers at nodes indicate bootstrap support values (100 replications). Gene names are color coded to indicate the major phylogenetic groups: archaea, purple; bacteria, blue; opistokonts, brown; plants, green; stramenopiles, red.
(3.00 MB TIF)

Figure S18 PHYML analysis of genes with homology to the cysteine synthase domains of Ps109172 and Ps109175. Numbers at nodes indicate bootstrap support values (100 replications). Gene names are color coded to indicate the major phylogenetic groups: archaea, purple; bacteria, blue; opistokonts, brown; plants, green; stramenopiles, red.
(3.00 MB TIF)

Figure S19 PHYML analysis of genes with homology to the cystathionine gamma lyase domain of Ps109222. Numbers at nodes indicate bootstrap support values (100 replications). Gene names are color coded to indicate the major phylogenetic groups: archaea, purple; bacteria, blue; opistokonts, brown; plants, green; stramenopiles, red.
(3.00 MB TIF)

Figure S20 PHYML analysis of genes with homology to the cystathionine beta lyase domain of Ps120833, a key step in the biosynthesis of methionine. Numbers at nodes indicate bootstrap support values (100 replications). Gene names are color coded to indicate the major phylogenetic groups: archaea, purple; bacteria, blue; opistokonts, brown; plants, green; stramenopiles, red.
(3.00 MB TIF)
