# Supplementary material for: Multiple Horizontal Gene Transfer Events and Domain Fusions Have Created Novel Regulatory and Metabolic Networks in the Oomycete Genome
Source: PLoS One. 2009 Jul 2;4(7):e6133. doi: 10.1371/journal.pone.0006133 (PMC2705460; doi:10.1371/journal.pone.0006133)
Supplement: Table S2 — Candidate Rosetta Stone proteins in the P sojae genome as identified by RSD analysis. This table identifies all the multifunctional gene models in P sojae that had orthologs to at least two of their domains in one or more species (0.16 MB DOC) [file pone.0006133.s002.doc]

Table S2. Candidate Rosetta Stone proteins in the *P sojae* genome as identified by RSD analysis

| P sojae gene | Species | | AGene Id | Function | A-score | B-Gene Id | Function | B-score | C-Gene Id | Function | C-Score |
| --- | --- | --- | --- | --- | --- | --- | --- | --- | --- | --- | --- |
| Ps112102 | *A_gossypii* | Q75DL7 | | Adenyl-sulfate | 1.2279 | Q74ZF6 | ATP- | 3.8614 |  | pyrrophosphatase |  |
|  | *A_thaliana* | Q9SRW7 | | kinase | 1.3006 | Q9LIK9 | sulfuryase | 0.9112 |  |  |  |
|  | *C_glabrata* | Q6FLM3 | |  | 1.0458 | Q6FXQ8 |  | 3.2528 |  |  |  |
|  | *K_lactis* | Q6CVB5 | |  | 0.9963 | Q6CNU6 |  | 3.5098 |  |  |  |
|  | *O_sativa* | 07g385601 | |  | 2.0136 | 04g020501 |  | 1.0201 |  |  |  |
|  | *P_patens* | Pp225860 | |  | 1.1201 | Pp140355 |  | 0.9815 |  |  |  |
|  | *P_stipitis* | Pis90976 | |  | 0.99 | Pis75451 |  | 3.5266 |  |  |  |
|  | *S_cerevisiae* | Q02196 | |  | 1.1107 | P08536 |  | 3.4148 |  |  |  |
|  | *S_pombe* | Q9P7G9 | |  | 1.0368 | P78937 |  | 3.0184 |  |  |  |
|  | *V_vinifera* | G_P00012057001 | |  | 1.1579 | G_P00014451001 |  | 0.9351 |  |  |  |
|  | *Synechococcus* |  | |  |  | syn_79420295 |  | 3.0625 | syn_79421383 |  | 2.4875 |
|  | *Nostoc sp* |  | |  |  |  |  |  | Nsp17132703 |  | 2.9138 |
|  |  |  | |  |  |  |  |  |  |  |  |
|  |  |  | |  |  |  |  |  |  |  |  |
| Ps128154 | *E. huxlevi* | Eh448559 | |  | 1.0209 | Eh435434 |  | 1.1525 |  |  |  |
|  |  |  | |  |  |  |  |  |  |  |  |
| Ps129035 | *A_thaliana* | Q9SSE7 | | Prephenate | 1.1472 | Q3E6N9 | prephenaste | 1.4339 | Q9SIE1 |  | 1.4428 |
|  | *B_japonicum* | Bj27376532 | | dehydratase | 1.7636 | Bj27382527 | dehydrogenase | 1.6889 |  |  |  |
|  | *O_sativa* | 03g177301 | |  | 1.0144 | 01g650902 |  | 1.2941 |  |  |  |
|  |  |  | |  |  |  |  |  |  |  |  |
| Ps129310 | *A_Gambiae* | Q7QH71 | | Zinc Finger | 2.4596 | Q5TYD1 | Gaf Domain | 1.9286 |  |  |  |
|  |  |  | |  |  |  |  |  |  |  |  |
| Ps129468 | *B_rerio* | Q6DH18 | | Syanptogamin | 3.7233 |  |  |  | Q6PC34 | EF-Hand | 2.3474 |
|  | *D_pseudoobscura* | Q2M189 | |  | 4.0568 |  |  |  | Q29IP4 |  | 2.4011 |
|  | *C_briggsae* | A8XLH0 | |  | 2.8996 |  |  |  | A8WYJ2 |  | 2.8554 |
|  |  |  | |  |  |  |  |  |  |  |  |
| Ps129786 | *A_gambiae* | Q7QI56 | |  | 2.8389 | Q7QIT3 |  | 3.9621 |  |  |  |
|  |  |  | |  |  |  |  |  |  |  |  |
| Ps130660 | *R. norvegicus* | ENSRNOP00000054580 | |  | 4.9052 | ENSRNOP00000038223 |  | 5.4568 |  |  |  |
|  |  |  | |  |  |  |  |  |  |  |  |
| Ps131776 | *A_gambiae* | Q7Q2S4 | | glycerol | 2.3745 | Q7PYF0 | glycerol | 2.8614 |  |  |  |
|  | *A egyptii* | Q172Q7Q172Q7_AEDAE | |  | 2.1788 | Q16L82Q16L82_AEDAE |  | 3.1881 |  |  |  |
|  | *A_fumigatus* | Q4WLB2 | | acyl reductase | 2.8641 | Q4WV69 | acyltransferase | 2.3612 |  |  |  |
|  | *C_briggsae* | Q622T8 | |  | 2.2974 | Q60Y13 |  | 2.1677 |  |  |  |
|  | *C_elegans* | Q9TZL9 | |  | 2.3831 | Q9U2E8 |  | 2.8798 |  |  |  |
|  | *D_melanogaster* | Q9V7S1 | |  | 2.1204 | Q8IMM7 |  | 3.0508 |  |  |  |
|  | *H_sapiens* | Q8WVX9 | |  | 1.9213 | Q5TBH6 |  | 1.2961 |  |  |  |
|  | *L_major* | Q4QAG7 | |  | 3.2051 | Q4Q396 |  | 2.7159 |  |  |  |
|  | *M_grisea* | MGG_02787 | |  | 2.7074 | MGG_01894 |  | 2.4746 |  |  |  |
|  | *M_musculus* | Q9D0Q1 | |  | 1.8366 | P98192 |  | 2.4762 |  |  |  |
|  | *P_tetraurelia* | GSPATP00011144001 | |  | 1.5695 | GSPATP00011528001 |  | 2.1049 |  |  |  |
|  | *R_norvegicus* | Q66H50 | |  | 2.4033 | Q9ES71 |  | 2.4708 |  |  |  |
|  | *T_nigroviridis* | Q4TCI8 | |  | 1.8326 | Q4RF56 |  | 2.6235 |  |  |  |
|  |  |  | |  |  |  |  |  |  |  |  |
| Ps132853 | *P tetraurelia* | GSPATP00031039001 | |  | 1.2991 | GSPATP00001903001 |  | 1.6032 |  |  |  |
|  |  |  | |  |  |  |  |  |  |  |  |
| Ps133120 | *B_japonicum* | Bj27377723 | | Diaminopimelate aminotransferase | 2.6427 | Bj27375796 | Dihydrodipicolinate reductase | 3.0517 |  |  |  |
|  |  |  | |  |  |  |  |  |  |  |  |
| Ps133512 | *D_discoideum* | Q7KWK0 | |  | 3.8222 | Q54DA2 |  | 2.7043 |  |  |  |
|  |  |  | |  |  |  |  |  |  |  |  |
| Ps133601 | *P tetraurelia* | GSPATP00006307001 | |  | 3.1095 | GSPATP00001485001 |  | 3.5685 |  |  |  |
|  |  |  | |  |  |  |  |  |  |  |  |
| Ps135760 | *H_sapiens* |  | |  |  | Q8TCT1 | phosphatase | 2.5247 | Q8TCD6 | phosphatase | 2.3525 |
|  | *P tetaurelia* |  | |  |  | GSPATP00036091001 |  | 2.5748 | GSPATP00033083001 |  | 2.6308 |
|  |  |  | |  |  |  |  |  |  |  |  |
| Ps136186 | *P_patens* | Pp164085 | |  | 5.1288 | Pp234441 |  | 3.9125 |  |  |  |
|  |  |  | |  |  |  |  |  |  |  |  |
| Ps136410 | *A_thaliana* | AT5G13280.1 | |  | 2.9048 | AT4G19710.2 |  | 2.6986 |  |  |  |
|  | *A_fumigatus* | Q4WTP2 | |  | 4.1511 | Q4WY36 |  | 3.0725 |  |  |  |
|  | *O_sativa* | 01g703003 | |  | 2.5713 | 09g122901 |  | 2.594 |  |  |  |
|  | *V_vinifera* | GSVIVP00006183001 | |  | 2.8212 | GSVIVP00032075001 |  | 2.6743 |  |  |  |
|  | *Y_lippolytica* | Q6C9F1 | |  | 3.5306 | Q6CAP2 |  | 2.792 |  |  |  |
|  |  |  | |  |  |  |  |  |  |  |  |
| Ps136487 | *A_gambiae* | Q7QE57 | |  | 8.3942 | Q7QK56 |  | 1.1942 |  |  |  |
|  | *H_sapiens* | Q2TBF5 | | Phox-like | 4.2115 | P31749 | protein | 1.1267 |  |  |  |
|  | *M_musculus* | Q8C084 | |  | 4.0453 | Q6GSA6 | kinase | 1.1253 |  |  |  |
|  | *R norvegicus* | ENSRNOP00000032451 | |  | 3.4754 | ENSRNOP00000038369 |  | 1.1253 |  |  |  |
|  |  |  | |  |  |  |  |  |  |  |  |
| Ps137793 | *B_rerio* | Q6NW80 | | cysteine | 2.899 | Q6PCS2 | unknown | 4.138 |  |  |  |
|  | *O_sativa* | 04g337801 | | protease | 2.977 | 06g169001 |  | 4.643 |  |  |  |
|  |  |  | |  |  |  |  |  |  |  |  |
| Ps140465 | *H sapiens* | Q5T5J9 | | nephrocystin | 2.4187 | Q96BN1 | Calcium binding | 1.6057 |  |  |  |
|  |  |  | |  |  |  |  |  |  |  |  |
| Ps141796 | *A. gambiae* | Q7Q4U8 | | pleckstrin | 2.5977 | Q5T4R3 | sterol | 4.5807 |  |  |  |
|  | *D pseudobscura* | Q29PN3 | |  | 4.4349 | Q299T0 | binding | 5.0816 |  |  |  |
|  | *B_rerio* | ENSDARP_87988 | |  | 4.2152 | ENSDARP_13230 | protein | 4.0252 |  |  |  |
|  |  |  | |  |  |  |  |  |  |  |  |
| Ps141887 | *T nigroviridis* | GENSCAN00000025219 | | Myosin head | 1.6279 | GSC00000001184 | ankyrin | 2.7574 |  |  |  |
|  |  |  | |  |  |  |  |  |  |  |  |
| Ps142688 | *C_briggsae* | Q60SV8 | | serine | 1.3571 | Q60Q10 | serine | 4.5591 |  |  |  |
|  | E_huxleyi | Eh415674 | | biosynthesis | 1.6589 | Eh255026 | biosynthesis | 0.7026 |  |  |  |
|  |  |  | |  |  |  |  |  |  |  |  |
| Ps143651 | *O_sativa* |  | |  |  | 03g503301 | protein | 1.836 | 06g396001 | protein | 2.0468 |
|  | *V_vinifera* |  | |  |  | GSVIVP00024519001 | kinase | 1.9857 | GSVIVP0001 3784001 | phosphatase | 1.7504 |
|  |  |  | |  |  |  |  |  |  |  |  |
| Ps143965 | *A. fumigatus* | Q4WPR6 | |  | 4.276 | Q4WDF2 |  | 2.3348 |  |  |  |
|  | *E huxlevi* | Eh463138 | |  | 4.9082 | Eh66731 |  | 2.4117 |  |  |  |
|  | *P tetraurelia* | GSPATP00022389001 | |  | 3.6685 | GSPATP00006336001 |  | 1.9896 |  |  |  |
|  |  |  | |  |  |  |  |  |  |  |  |
| Ps144830 | *H_sapiens* | Q9NUW1 | | NRPS | 2.7675 | Q4L235 | NRPS | 3.8082 |  |  |  |
|  | *Y_pestris* | YPA3377 | |  | 3.6505 | YPA1289 |  | 2.7489 |  |  |  |
|  | *D_melanogaster* |  | |  | 3.3323 | Q76858 |  | 3.3 | Q9VG87 |  | 2.6693 |
|  | *D pseudoobscura* |  | |  |  | Q29B08 |  | 3.3089 | Q29B58 |  | 2.724 |
|  | *E huxlevi* | Eh466966 | |  | 3.6085 |  |  |  | Eh454037 |  | 1.3886 |
|  | *Nostoc* | Nsp17131136 | |  | 2.4062 | Nsp17131741 |  | 1.7858 | Nsp17134496 |  | 1.279 |
|  | *T_nigroviridis* | Q4TBP1 | |  | 2.2361 |  |  |  | Q4SAB7 |  | 2.8684 |
|  |  |  | |  |  |  |  |  |  |  |  |
| Ps156997 | *B_pennsylvanicus* | Q493N2 | | Phosphoadenosine | 1.7187 | Q491X8 | Glutaredoxin | 2.4552 |  |  |  |
|  | *Nostoc* | Nsp17133600 | | phosphosulfate reductase | 2.1406 | Nsp17135361 |  | 2.4858 |  |  |  |
|  | *Y_pestris* | YPA2867 | |  | 1.6854 | YPA0618 |  | 1.4655 |  |  |  |
