# Supplementary figures and images for: Multiple Horizontal Gene Transfer Events and Domain Fusions Have Created Novel Regulatory and Metabolic Networks in the Oomycete Genome
Source: PLoS One. 2009 Jul 2;4(7):e6133. doi: 10.1371/journal.pone.0006133 (PMC2705460; doi:10.1371/journal.pone.0006133)

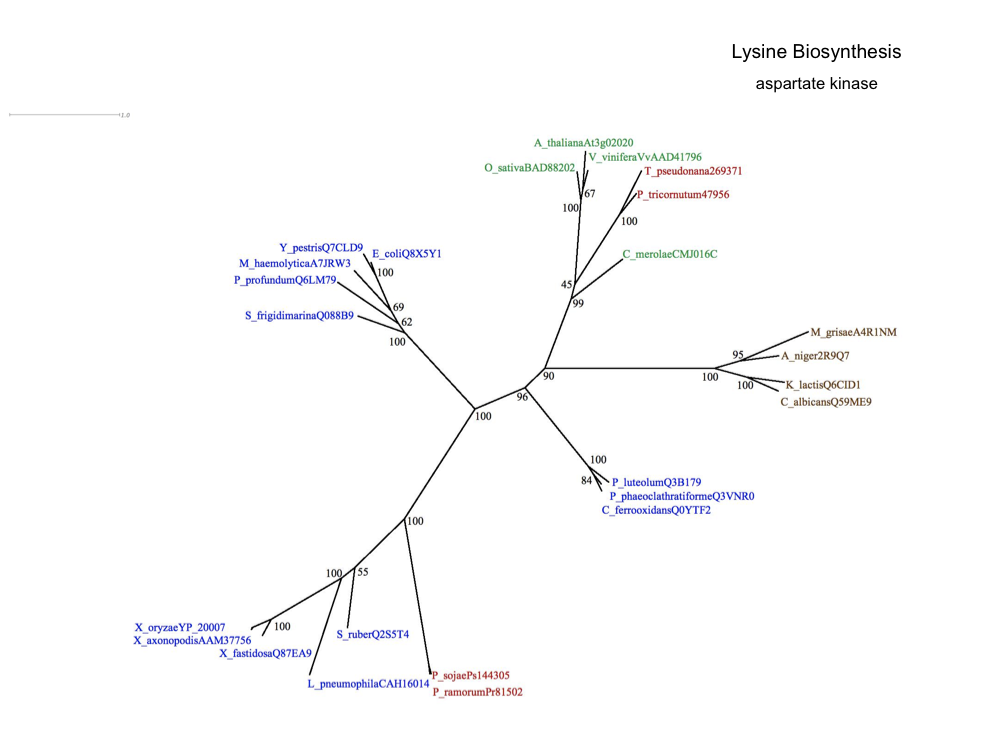

Supplement: Figure S1 — PHYML analysis of genes with homology to the aspartate kinase domain of Ps144305. The P. sojae enzyme is the first committed step in a predicted lysine biosynthetic pathway of oomycetes. Numbers at nodes indicate bootstrap support values (100 replications). Gene names are color coded to indicate the major phylogenetic groups: archaea, purple; bacteria, blue; opistokonts, brown; plants, green; stramenopiles, red. (3.00 MB TIF) [file pone.0006133.s004.tif]

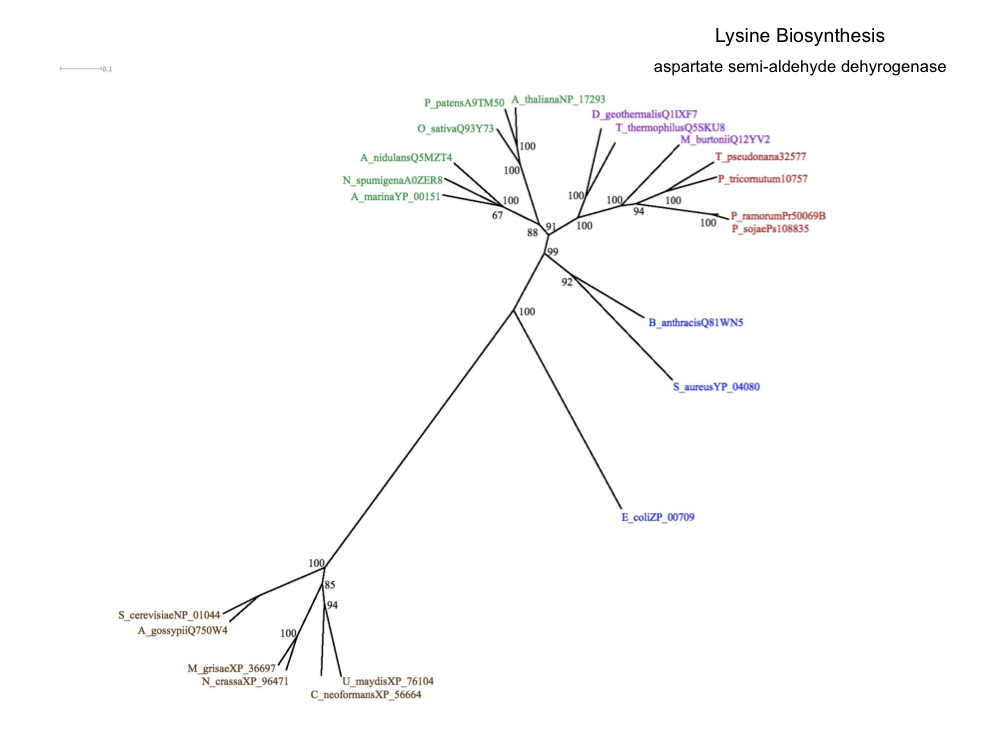

Supplement: Figure S2 — PHYML analysis of genes with homology to the aspartate semi-aldehyde dehydrogenase domain of Ps108835. The P. sojae enzyme is the second step a predicted lysine biosynthetic pathway of oomycetes. Numbers at nodes indicate bootstrap support values (100 replications). Gene names are color coded to indicate the major phylogenetic groups: archaea, purple; bacteria, blue; opistokonts, brown; plants, green; stramenopiles, red. (3.00 MB TIF) [file pone.0006133.s005.tif]

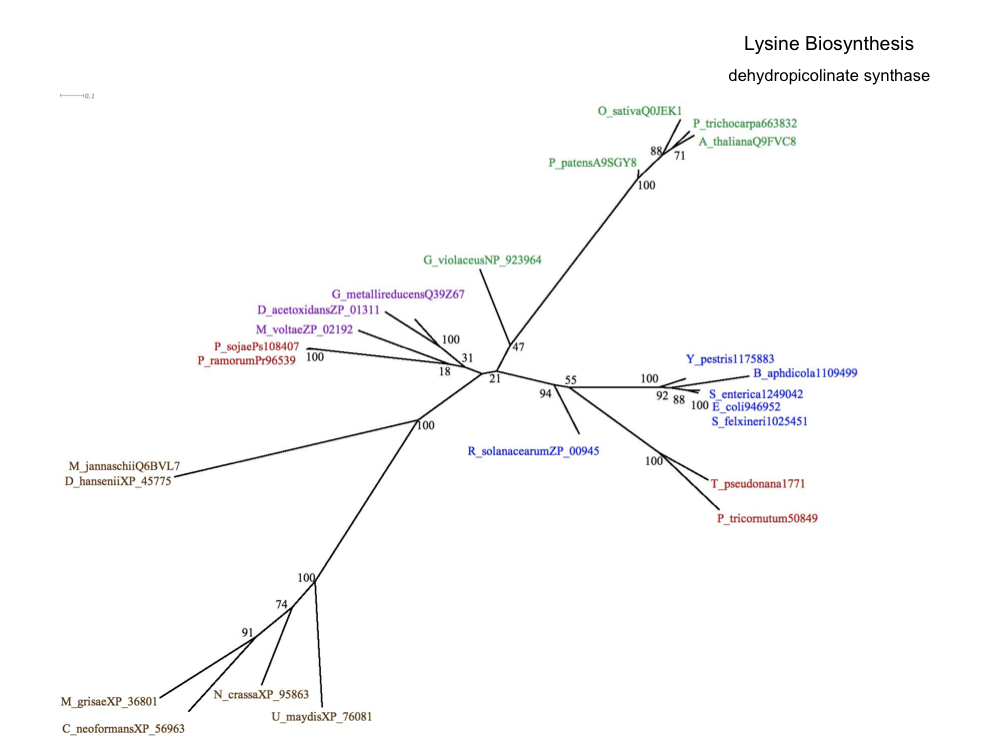

Supplement: Figure S3 — PHYML analysis of genes with homology to the dihydropicolinate synthase domain of Ps108407. The P. sojae enzyme is the third step in a predicted lysine biosynthetic pathway of oomycetes. Numbers at nodes indicate bootstrap support values (100 replications). Gene names are color coded to indicate the major phylogenetic groups: archaea, purple; bacteria, blue; opistokonts, brown; plants, green; stramenopiles, red. (3.00 MB TIF) [file pone.0006133.s006.tif]

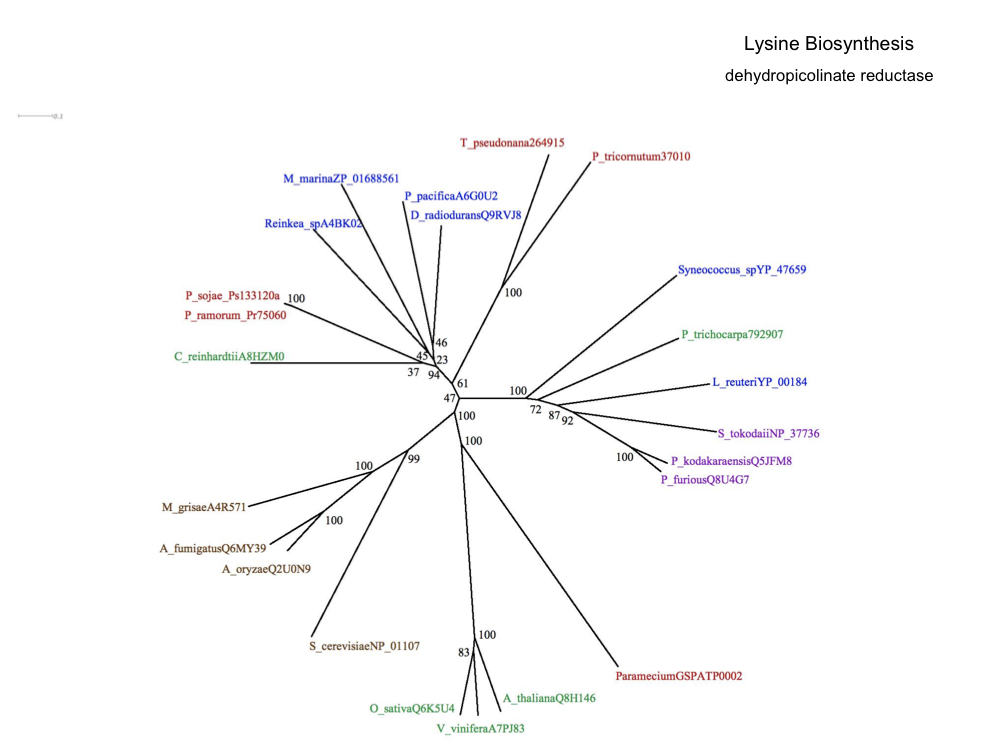

Supplement: Figure S4 — PHYML analysis of genes with homology to the dihydropicolinate reductase domain of Ps133120. The P sojae enzyme carries out step four and step five of a predicted lysine biosynthetic pathway of oomycetes. Numbers at nodes indicate bootstrap support values (100 replications). Gene names are color coded to indicate the major phylogenetic groups: archaea, purple; bacteria, blue; opistokonts, brown; plants, green; stramenopiles, red. (3.00 MB TIF) [file pone.0006133.s007.tif]

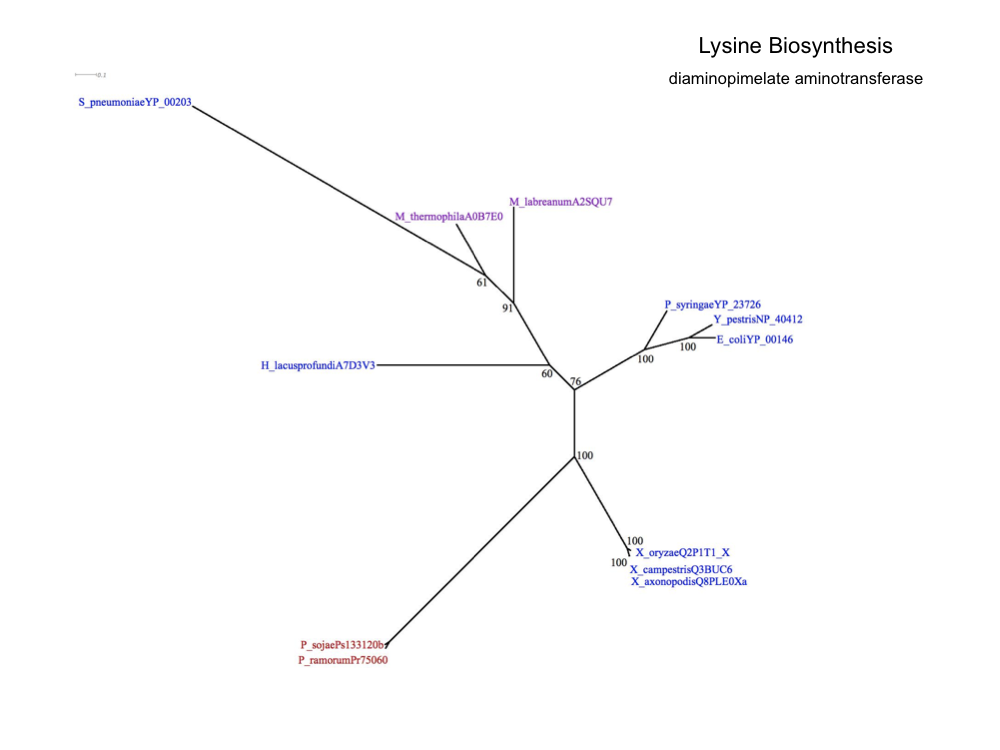

Supplement: Figure S5 — PHYML analysis of genes with homology to the diaminopimelate aminotransferase domain of Ps133120. The P sojae enzyme carries out step four and step five of a predicted lysine biosynthetic pathway of oomycetes. Numbers at nodes indicate bootstrap support values (100 replications). Gene names are color coded to indicate the major phylogenetic groups: archaea, purple; bacteria, blue; opistokonts, brown; plants, green; stramenopiles, red. (3.00 MB TIF) [file pone.0006133.s008.tif]

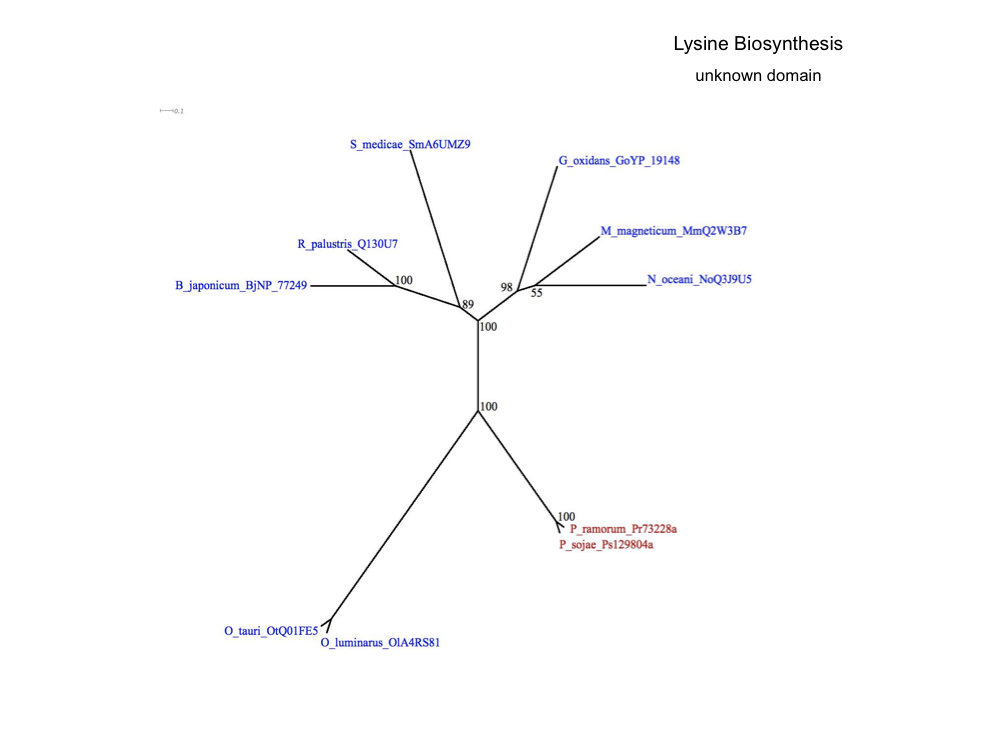

Supplement: Figure S6 — PHYML analysis of genes with homology to the N-terminal domain of Ps129804. Numbers at nodes indicate bootstrap support values (100 replications). Gene names are color coded to indicate the major phylogenetic groups: archaea, purple; bacteria, blue; opistokonts, brown; plants, green; stramenopiles, red (3.00 MB TIF) [file pone.0006133.s009.tif]

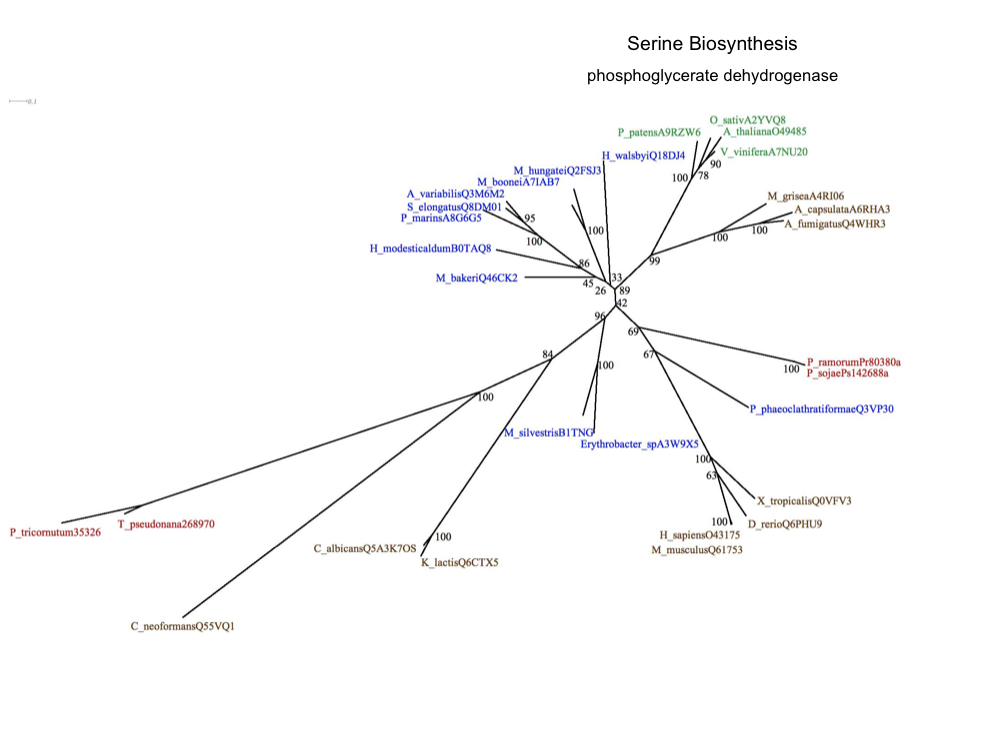

Supplement: Figure S7 — PHYML analysis of genes with homology to the phosphoglycerate dehydrogenase domain of Ps142688. The P. sojae enzyme contains domains which catalyze the first two steps in a serine biosynthetic pathway. Numbers at nodes indicate bootstrap support values (100 replications). Gene names are color coded to indicate the major phylogenetic groups: archaea, purple; bacteria, blue; opistokonts, brown; plants, green; stramenopiles, red. (3.00 MB TIF) [file pone.0006133.s010.tif]

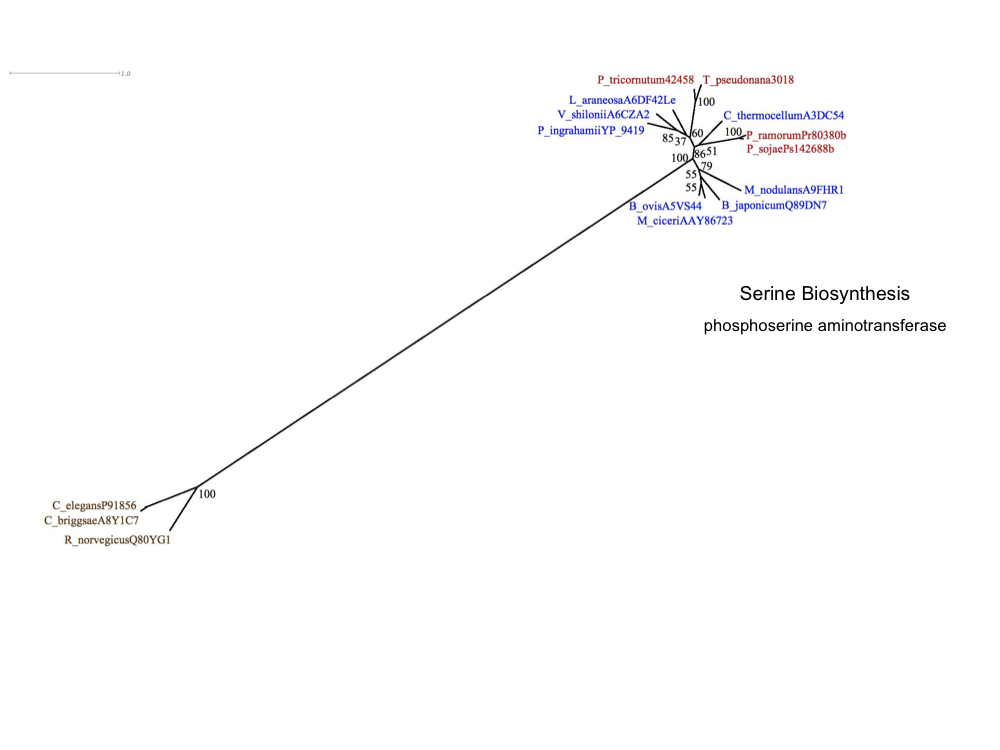

Supplement: Figure S8 — PHYML analysis of genes with homology to the phosphoserine aminotransferase domain of Ps142688. The P. sojae enzyme catalyzes the first two steps in a serine biosynthetic pathway. Numbers at nodes indicate bootstrap support values (100 replications). Note that the diatom genomes contain agene model in the same clade as oomycete genomes. Gene names are color coded to indicate the major phylogenetic groups: archaea, purple; bacteria, blue; opistokonts, brown; plants, green; stramenopiles, red. (3.00 MB TIF) [file pone.0006133.s011.tif]

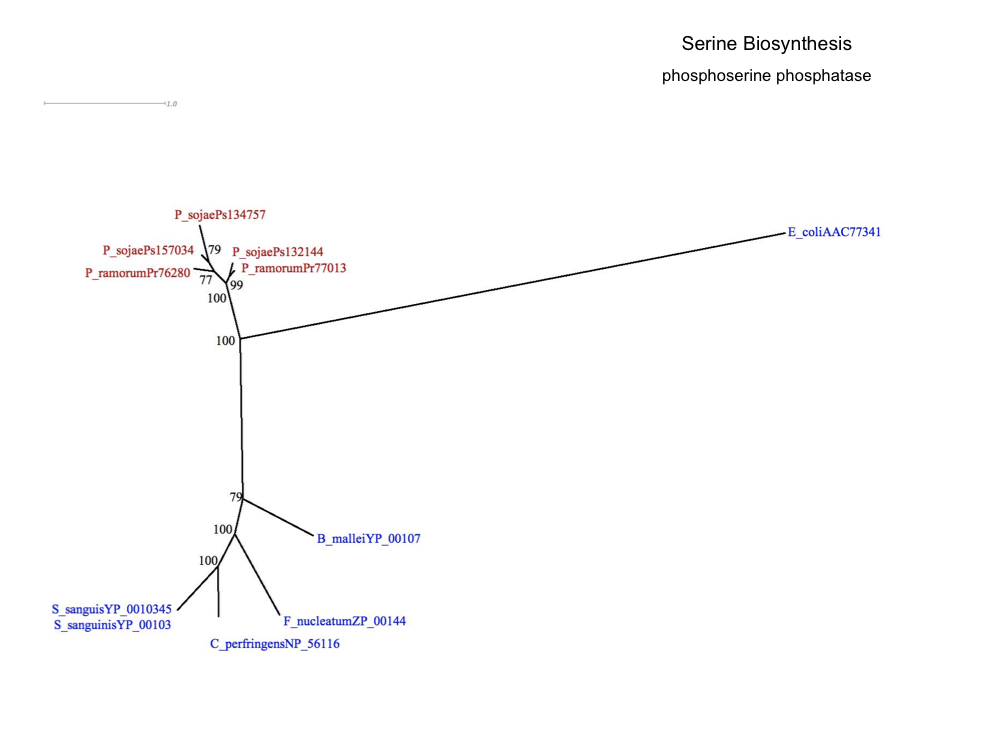

Supplement: Figure S9 — PHYML analysis of genes with homology to the phosphoserine phosphatase domians of Ps132144 Ps134157, and Ps157034. The P. sojae enzymes catalyze the last step in a serine biosynthetic pathway. Numbers at nodes indicate bootstrap support values (100 replications). Gene names are color coded to indicate the major phylogenetic groups: archaea, purple; bacteria, blue; opistokonts, brown; plants, green; stramenopiles, red. (3.00 MB TIF) [file pone.0006133.s012.tif]

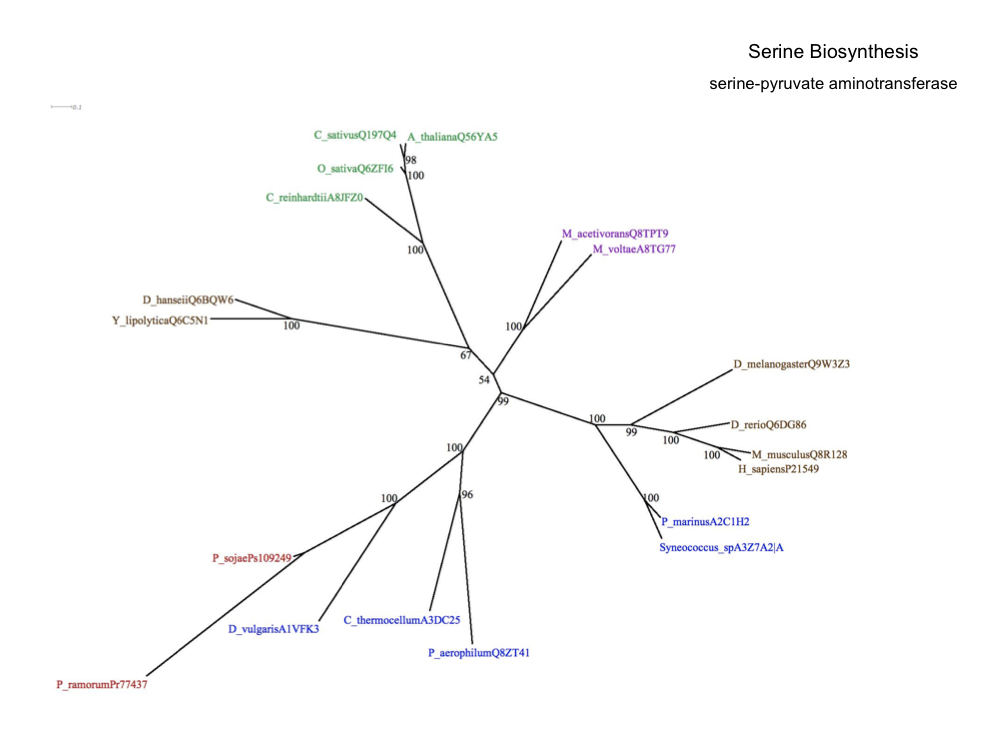

Supplement: Figure S10 — PHYML analysis of genes with homology to the serine-pyruvate aminotransferase domain of P sojae. The P. sojae enzyme catalyzes the synthesis of serine from pyruvate, a biosynthetic strategy independent of the P. sojae enzymes analyzed in Figs. S7, S8, S9. Numbers at nodes indicate bootstrap support values (100 replications). Gene names are color coded to indicate the major phylogenetic groups: archaea, purple; bacteria, blue; opistokonts, brown; plants, green; stramenopiles, red. (3.00 MB TIF) [file pone.0006133.s013.tif]

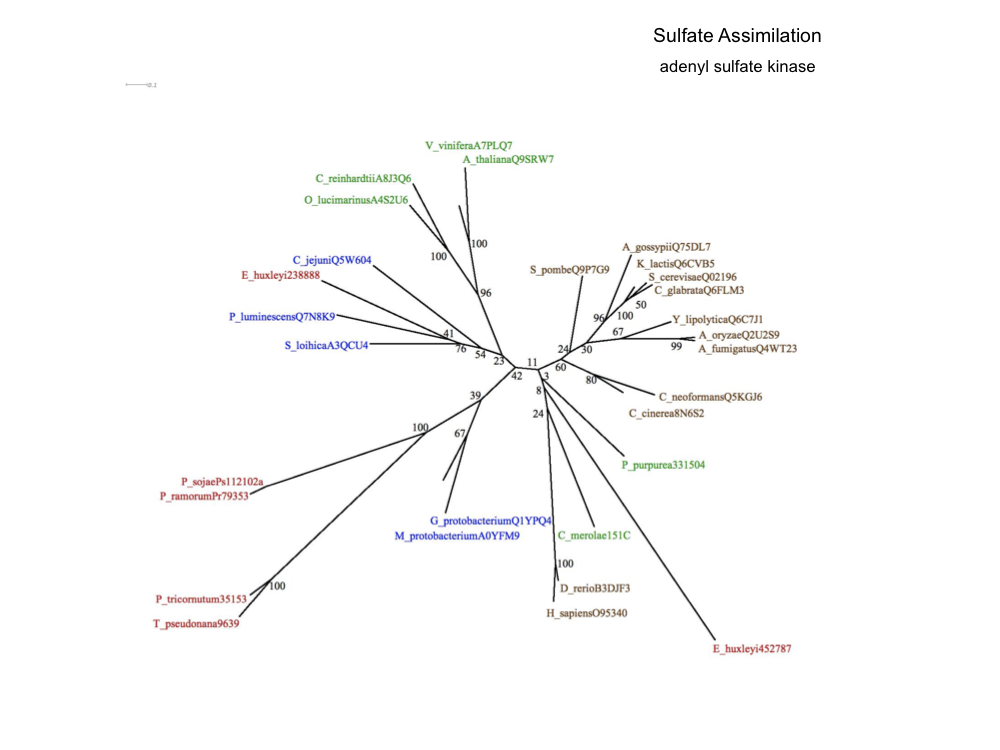

Supplement: Figure S11 — PHYML analysis of genes with homology to the adenylsulfate kinase domain of Ps112102. Numbers at nodes indicate bootstrap support values (100 replications). The oomycete, diatoms and E. huxleyi genomes contain a single protein with adenylsulfate kinase, ATP sulfurylase and pyrrophosphatase domains. The domains of these proteins have been subjected to separate phylogenetic analyses to determine whether they share a common phylogenetic origin. Gene names are color coded to indicate the major phylogenetic groups: archaea, purple; bacteria, blue; opistokonts, brown; plants, green; stramenopiles, red. (3.00 MB TIF) [file pone.0006133.s014.tif]

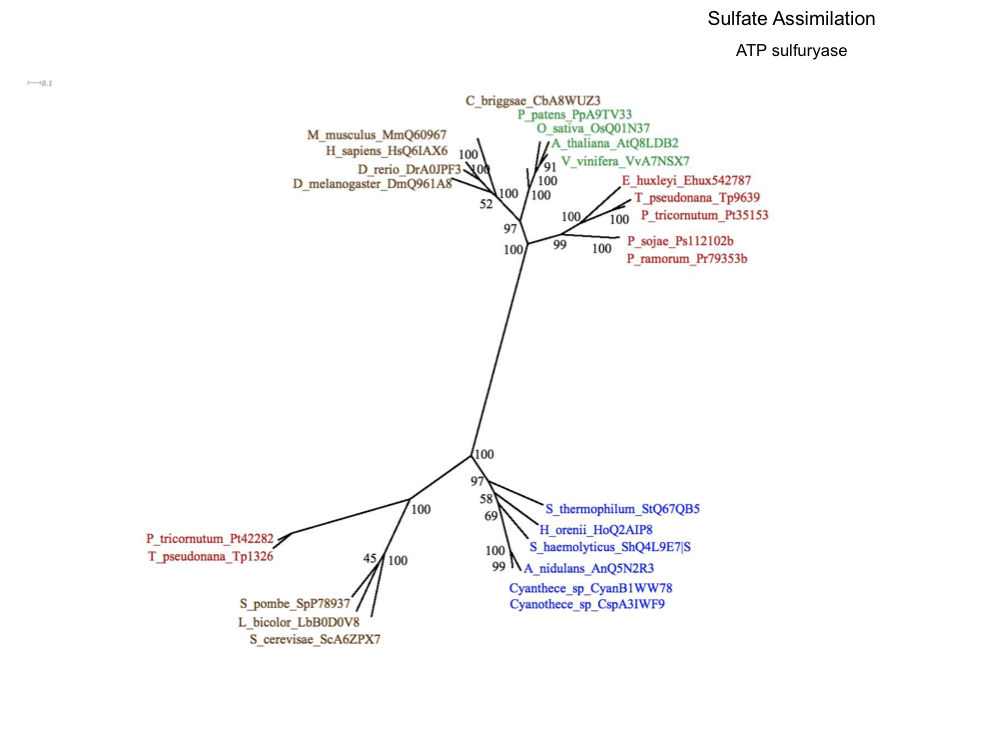

Supplement: Figure S12 — PHYML analysis of genes with homology to the ATP sulfurylase domain of Ps112102. Numbers at nodes indicate bootstrap support values (100 replications). The oomycete, diatoms and E. huxleyi genomes contain a single protein with adenylsulfate kinase, ATP sulfurylase and pyrrophosphatase domains. The domains of these proteins have been subjected to separate phylogenetic analyses to determine whether they share a common phylogenetic origin. Gene names are color coded to indicate the major phylogenetic groups: archaea, purple; bacteria, blue; opistokonts, brown; plants, green; stramenopiles, red. (3.00 MB TIF) [file pone.0006133.s015.tif]

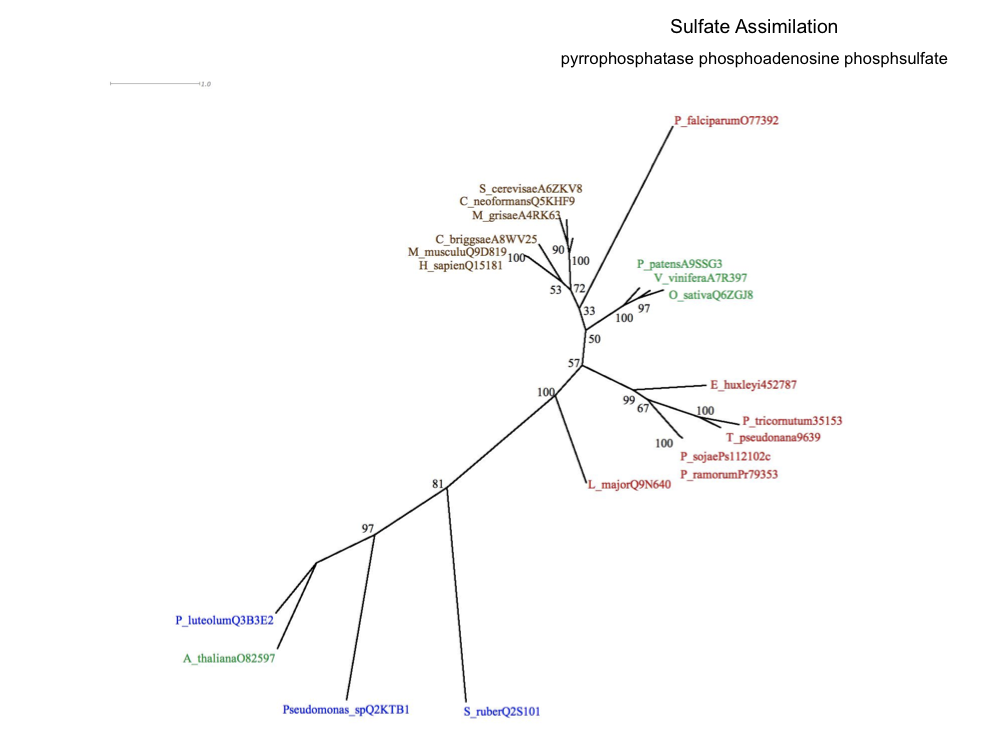

Supplement: Figure S13 — PHYML analysis of genes with homology to the pyrrophosphatase domain of Ps112102. Numbers at nodes indicate bootstrap support values (100 replications). The oomycete, diatoms and E. huxleyi genomes contain a single protein with adenylsulfate kinase, ATP sulfurylase and pyrrophosphatase domains. The domains of these proteins have been subjected to separate phylogenetic analyses to determine whether they share a common phylogenetic origin. Gene names are color coded to indicate the major phylogenetic groups: archaea, purple; bacteria, blue; opistokonts, brown; plants, green; stramenopiles, red. (3.00 MB TIF) [file pone.0006133.s016.tif]

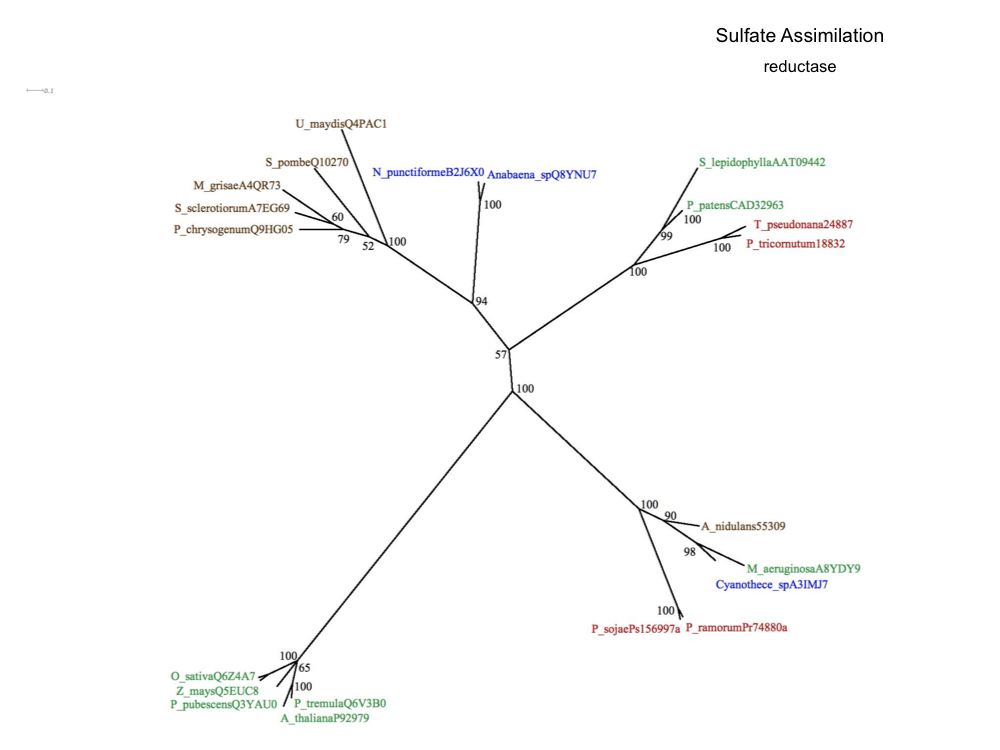

Supplement: Figure S14 — PHYML analysis of genes with homology to the phosphoadenosine phosphosulfate reductase domain of Ps156997. The P sojae enzyme catalyzes step four in the sulfate assimilation pathway. Numbers at nodes indicate bootstrap support values (100 replications). Gene names are color coded to indicate the major phylogenetic groups: archaea, purple; bacteria, blue; opistokonts, brown; plants, green; stramenopiles, red. (3.00 MB TIF) [file pone.0006133.s017.tif]

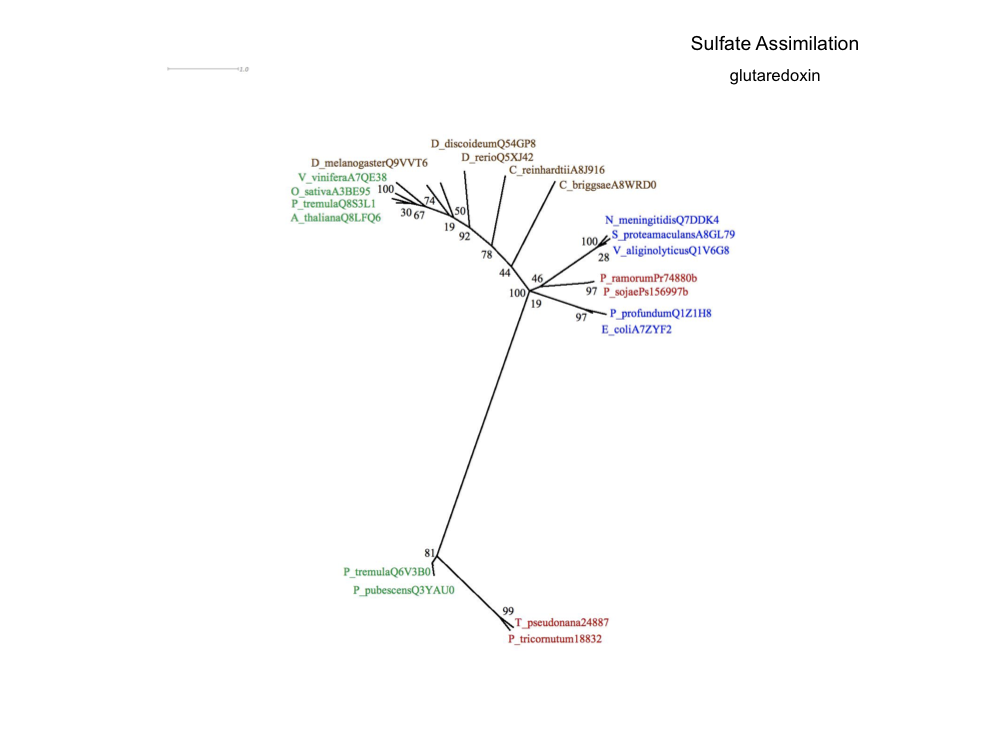

Supplement: Figure S15 — PHYML analysis of genes with homology to a novel glutaredoxin domain on Ps156997. Numbers at nodes indicate bootstrap support values (100 replications). Gene names are color coded to indicate the major phylogenetic groups: archaea, purple; bacteria, blue; opistokonts, brown; plants, green; stramenopiles, red. (3.00 MB TIF) [file pone.0006133.s018.tif]

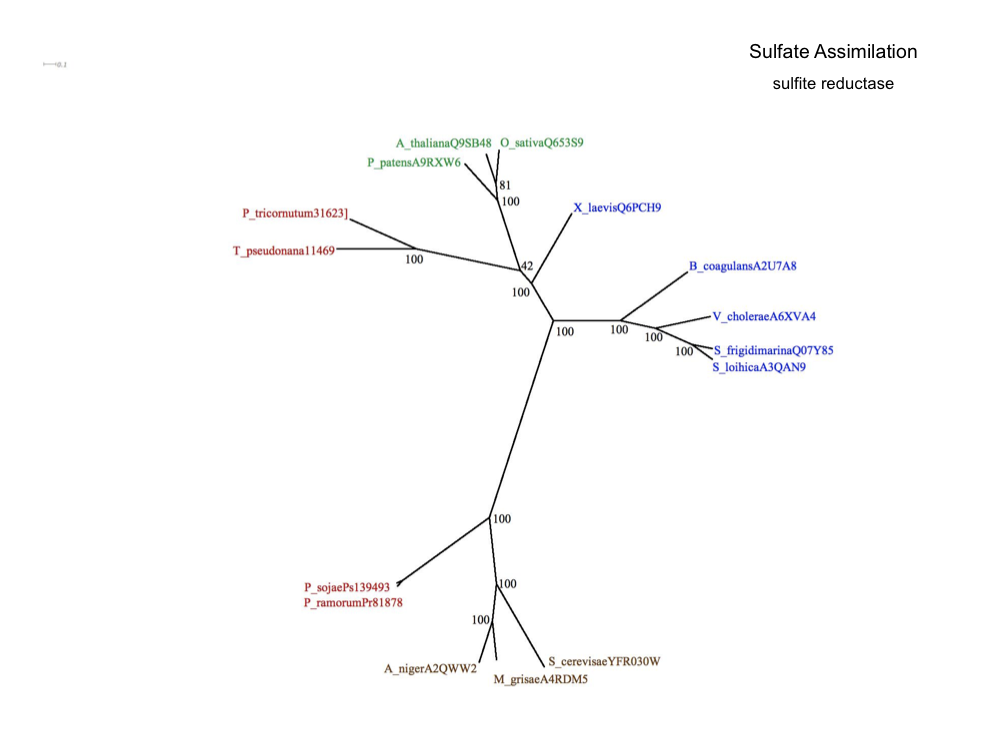

Supplement: Figure S16 — PHYML analysis of genes with homology to Ps139493. The P. sojae enzyme is predicted to form a complex with Ps139488 to catalyze the reduction of sulfite. Numbers at nodes indicate bootstrap support values (100 replications). Gene names are color coded to indicate the major phylogenetic groups: archaea, purple; bacteria, blue; opistokonts, brown; plants, green; stramenopiles, red. (3.00 MB TIF) [file pone.0006133.s019.tif]

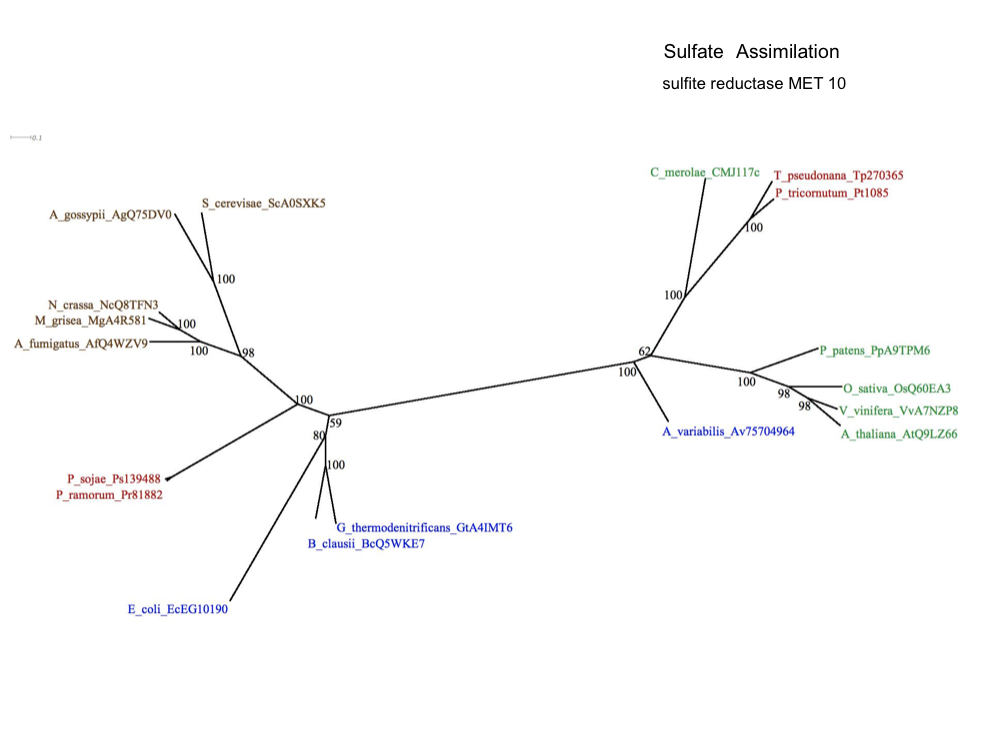

Supplement: Figure S17 — PHYML analysis of genes with homology to Ps139488. The P. sojae enzyme is predicted to form a complex with Ps139493 to catalyze the reduction of sulfite. Numbers at nodes indicate bootstrap support values (100 replications). Gene names are color coded to indicate the major phylogenetic groups: archaea, purple; bacteria, blue; opistokonts, brown; plants, green; stramenopiles, red. (3.00 MB TIF) [file pone.0006133.s020.tif]

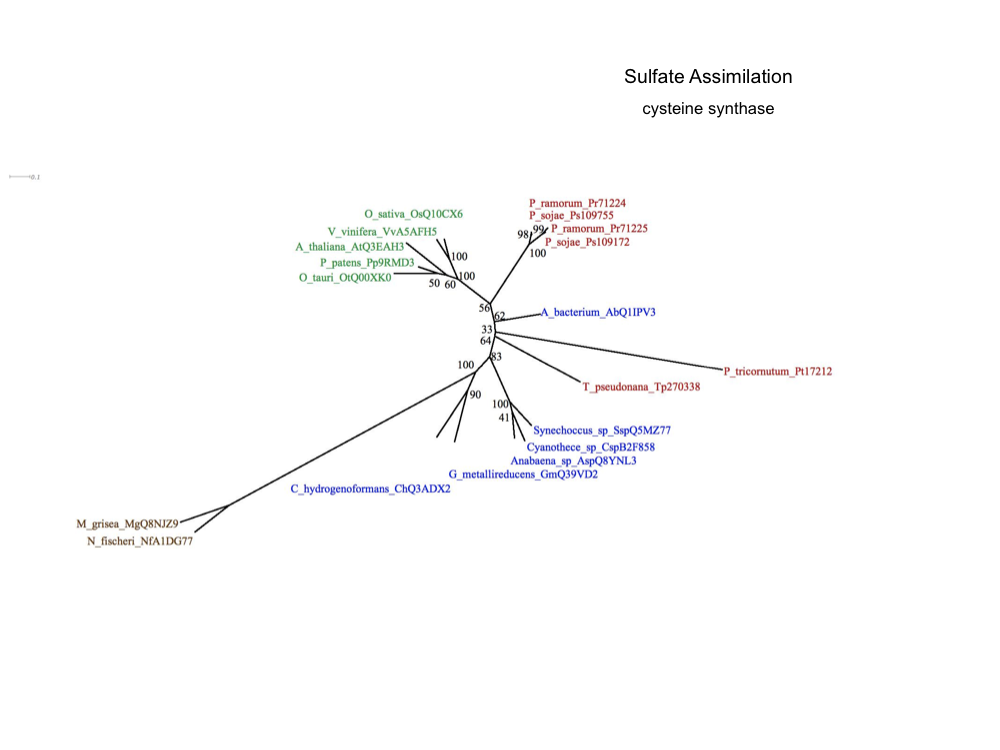

Supplement: Figure S18 — PHYML analysis of genes with homology to the cysteine synthase domains of Ps109172 and Ps109175. Numbers at nodes indicate bootstrap support values (100 replications). Gene names are color coded to indicate the major phylogenetic groups: archaea, purple; bacteria, blue; opistokonts, brown; plants, green; stramenopiles, red. (3.00 MB TIF) [file pone.0006133.s021.tif]

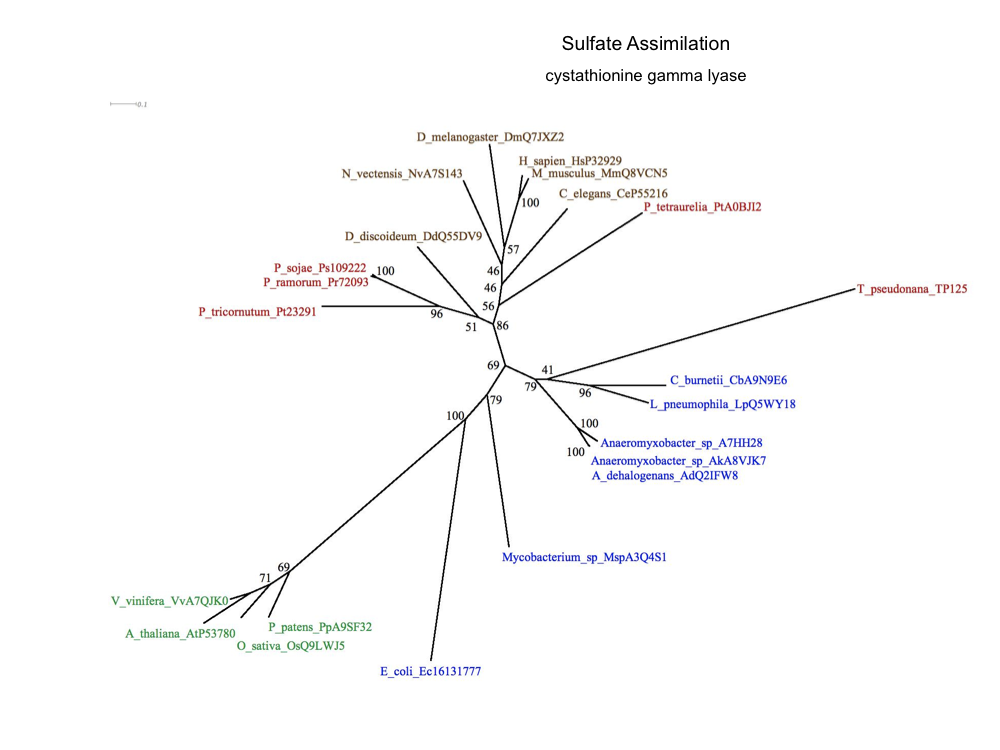

Supplement: Figure S19 — PHYML analysis of genes with homology to the cystathionine gamma lyase domain of Ps109222. Numbers at nodes indicate bootstrap support values (100 replications). Gene names are color coded to indicate the major phylogenetic groups: archaea, purple; bacteria, blue; opistokonts, brown; plants, green; stramenopiles, red. (3.00 MB TIF) [file pone.0006133.s022.tif]

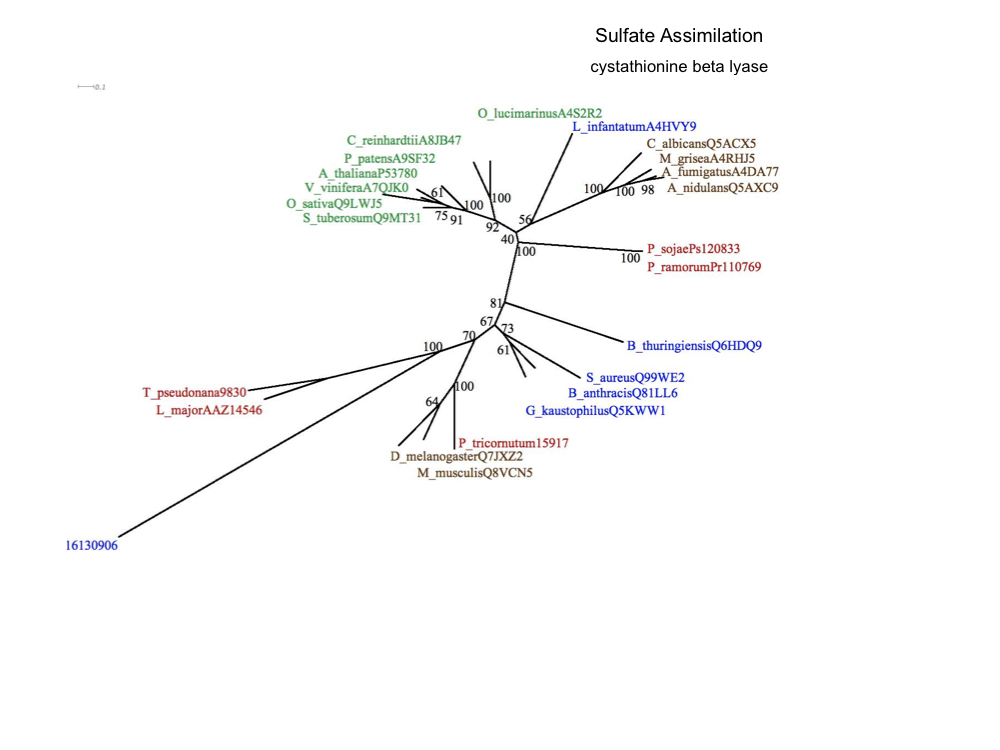

Supplement: Figure S20 — PHYML analysis of genes with homology to the cystathionine beta lyase domain of Ps120833, a key step in the biosynthesis of methionine. Numbers at nodes indicate bootstrap support values (100 replications). Gene names are color coded to indicate the major phylogenetic groups: archaea, purple; bacteria, blue; opistokonts, brown; plants, green; stramenopiles, red. (3.00 MB TIF) [file pone.0006133.s023.tif]
